# Application of *Lacticaseibacillus paracasei* and *Tetragenococcus halophilus* as adjunct starter cultures in Gouda cheese production

**DOI:** 10.3389/fmicb.2025.1719725

**Published:** 2026-01-16

**Authors:** Hannes Decadt, Stefan Weckx, Luc De Vuyst

**Affiliations:** Research Group of Industrial Microbiology and Food Biotechnology (IMDO), Faculty of Sciences and Bioengineering Sciences, Vrije Universiteit Brussel, Brussels, Belgium

**Keywords:** adjunct starter cultures, amino acids, biogenic amines, high-throughput full-length 16S rRNA gene sequencing, pilot-scale production, starter culture selection

## Abstract

Adjunct starter cultures are of interest in pasteurized cheese production as they provide additional flavor, ensure reproducible quality, and enable faster ripening. In the current study, two adjunct culture strains were selected, namely *Lacticaseibacillus paracasei* LP46, selected out of 49 isolates from mature Gouda cheese, and *Tetragenococcus halophilus* TH63, selected out of 244 isolates from a Gouda cheese brine. To date, the use of a *Tetragenococcus* strain in cheese production has not been reported. Both strains were applied in pilot-scale Gouda cheese productions, and the cheeses were investigated up to 32 weeks of ripening by a multiphasic approach encompassing culture-dependent and culture-independent microbiological analysis, meta-metabolomics, and organoleptic evaluations. In the case of the *Lacc. paracasei* strain, three batches with each time a different primary starter culture mixture were produced to investigate the effect of the primary starter culture mixture on the adjunct starter culture. The adjunct starter culture strains were able to become abundant in the cheeses. Furthermore, both adjunct starter culture strains seemed to repress the *Leuconostoc* strains from the primary starter culture mixtures. The *Lacc. paracasei* adjunct starter culture was associated with higher concentrations of acetoin and cadaverine, despite showing no biogenic amine production during the screening process. The *T. halophilus* adjunct starter culture increased the total amino acid concentration by 55% and also resulted in higher concentrations of acetoin and 2,3-butanedione. However, the organoleptic evaluation could not indicate a significant difference between the negative controls and the cheeses with adjunct starter cultures. Nevertheless, this first application of a *Tetragenococcus* strain in Gouda cheese showed that *T. halophilus* seems a promising cheese adjunct starter culture.

## 1 Introduction

For ages, the production of cheese relies on the use of adventitious microorganisms, a practice that over time evolved from spontaneous milk fermentation into backslopping, during which a part of a previous production batch was used as inoculum for the new production batch to ensure its quality (Bassi et al., 2015). Currently, backslopping is still applied in the case of Italian cheeses, such as Parmigiano Reggiano and Grana Padano, whereby whey of a previous production batch is used as a natural whey starter, of which the microbial composition can vary over time (Sola et al., 2022). However, most other cheeses are produced by the application of a defined starter culture (DSC), which is composed of one or a few well-known strains, resulting in highly reproducible cheese production batches. Alternatively, Gouda cheeses are made with a mesophilic undefined starter culture (USC) containing a mixture of strains of *Lactococcus lactis*, *Lactococcus lactis* biovar diacetylactis, and *Lactococcus cremoris*, and (a) strain(s) of one or more *Leuconostoc* species (Decadt and De Vuyst, 2023). These USCs originate from domesticated inoculum mixtures applied in cheese plants or farms for a long time, and are propagated as little as possible to safeguard the original composition (Smid et al., 2014). The advantage of such USCs is their robustness against bacteriophage attacks compared with DSCs (Erkus et al., 2013; Smid et al., 2014). Despite this robustness, industrial Gouda cheese producers still apply a rotation strategy with different USCs to avoid phage infections, which in turn might result in batch-to-batch variations (Decadt et al., 2023).

Nowadays, most Gouda cheeses are made from pasteurized milk to reduce the variable adventitious microorganisms and to avoid pathogens (Düsterhöft et al., 2017). However, consumers mostly describe the flavor of raw-milk cheeses as richer and more intense than pasteurized milk cheeses (Montel et al., 2014). To benefit from the positive contributions that the raw-milk microbiota can bring to the cheese flavor, adjunct starter cultures (adjuncts) are often added, in addition to the DSCs or USCs, during the production of pasteurized milk cheeses (Irlinger et al., 2017). These adjuncts guarantee a reproducible, desired flavor and can also suppress the growth of undesired non-starter lactic acid bacteria (NSLAB). When adjuncts are used, the DSCs or USCs are referred to as primary starter cultures, as their main role is to rapidly acidify the milk, whereas the adjuncts fulfill their role during the cheese ripening phase. Mostly, the species used as adjuncts for Gouda cheese production are part of the desired NSLAB, such as *Lacticaseibacillus (para)casei*, *Lacticaseibacillus rhamnosus*, *Lactobacillus helveticus*, and *Latilactobacillus sakei* (Decadt and De Vuyst, 2023). Especially *Lacticaseibacillus paracasei*, which is the most encountered NSLAB species in Gouda cheese, has been applied in several Gouda cheese productions, mostly resulting in an increased proteolysis (Asahina et al., 2020; Bielecka and Cichosz, 2017; Porcellato et al., 2013; Saiki et al., 2018; Van Hoorde et al., 2010). Since the concentration of free amino acids is a main determinant of cheese age (Decadt et al., 2024a), adjuncts such as *Lacc. paracasei* might accelerate cheese ripening. However, the addition of *Lacc. paracasei* can sometimes reduce the growth of leuconostocs from the primary starter culture (Porcellato et al., 2013). Hence, the effects of an adjunct might also be dependent on the primary starter culture used.

The application of culture-independent high-throughput sequencing (HTS) has revealed the presence of several bacteria not previously found in cheeses (Quigley et al., 2012), such as halophilic lactic acid bacteria (HLAB). Simultaneously, salt-enriched agar media are increasingly applied in recent years for cheese studies, showing the presence of several HLAB species, such as *Marinilactibacillus psychrotolerans*, *Tetragenococcus halophilus*, *Tetragenococcus koreensis*, and *Weissella thailandensis* (Ishikawa et al., 2007; Morales et al., 2011; Rodríguez et al., 2022; Unno et al., 2020). These HLAB species are an underexplored pool of possible adjuncts. Based on phenotypic and genomic characterization, especially *T. halophilus* has been suggested as a candidate adjunct (Morales et al., 2011; Rodríguez et al., 2022). This species has a history of safe use in salty, oriental fermented foods, such as soy sauce, fish sauce, and shrimp paste (Justé et al., 2014). Moreover, it is abundantly present in cheese brines (Haastrup et al., 2018; Marino et al., 2017; Vermote et al., 2018) and, therefore, often found in Gouda cheeses (Decadt et al., 2023, 2024a; Decadt et al., 2024b). Yet, to the best of the authors’ knowledge, *T. halophilus* has never been reported to be applied as an adjunct in any cheese production. However, since this species has occasionally been linked with histamine production (Botello-Morte et al., 2022), screening for biogenic amine production is of utmost importance when considering it as an adjunct.

The current study aimed to screen isolates of *Lacc. paracasei* and *T. halophilus*, previously collected from Gouda cheese production batches, including brine and rennet samples, to select a suitable strain for use as an adjunct in Gouda cheese production. Additionally, the *Lacc. paracasei* adjunct strain was combined with three different commercial primary Gouda starter culture mixtures to investigate possible interactions between the primary starter culture and the adjunct. A multiphasic approach was applied to investigate all cheese production batches at different time points, from the milk until the cheeses ripened for 32 weeks, encompassing culture-dependent and culture-independent microbiological analyses, meta-metabolomics, and organoleptic evaluations.

## 2 Materials and methods

### 2.1 Screening for and selection of adjunct starter culture strains

#### 2.1.1 Lacticaseibacillus paracasei

During a previous study, 49 isolates of *Lacc. paracasei* were obtained from Gouda cheeses ripened for 75 weeks (Decadt et al., 2023). These isolates, preserved in de Man-Rogosa-Sharpe (MRS) broth (Oxoid, Basingstoke, Hampshire, United Kingdom) supplemented with 25% (v/v) glycerol in cryovials at −80 °C, were streaked on MRS agar medium (Oxoid) and grown at 30 °C for 3 days. Afterward, single colonies were picked and inoculated in an appropriate medium to assess the proteolytic properties, the biogenic amine formation abilities, and the production of volatile organic compounds of their cultures through phenotypical tests (Randazzo et al., 2021).

To assess the proteolytic properties, each culture was grown overnight in tryptone-yeast extract-glucose (TYG) broth [containing 5.0 g/L of tryptone (Oxoid), 2.5 g/L of yeast extract (Oxoid), and 1.0 g/L of glucose (Merck, Darmstadt, Germany)] supplemented with skim milk powder (1%, m/m; Sigma-Aldrich, Saint Louis, Missouri, United States). Subsequently, the cultures were spotted on TYG agar medium [TYG broth with 15 g/L of agar (Oxoid)] supplemented with skim milk powder (1%, m/m; Sigma-Aldrich), and incubated at 30 °C for 4 days (Meng et al., 2018). The size of the clearing zone, being an indicator of the proteolysis degree, was then manually assessed. Additionally, cultures were inoculated (1%, v/v) in TYG broth supplemented with 15 g/L of casein (Sigma-Aldrich) and 5 g/L of NaCl (Merck), and incubated at 30 °C for 7 days. The same, not inoculated medium served as a negative control. Afterward, free amino acids were quantified using ultra-performance liquid chromatography coupled to tandem mass spectrometry (UPLC-MS/MS) as described below.

To assess biogenic amine production, each culture was first grown in MRS broth (Oxoid) supplemented with histidine, lysine, and ornithine (2.5 g/L each; all from VWR International, Darmstadt, Germany), pyridoxal-5-phosphate (0.05 g/L; Sigma-Aldrich), and tyrosine (5.0 g/L; Sigma-Aldrich), followed by inoculation (0.1%, v/v) in a liquid medium optimized for the production of biogenic amines defined before (Bover-Cid and Holzapfel, 1999), but without meat extract and supplemented with 5 g/L of NaCl (Merck) and incubation at 30 °C for 6 days. The same, not inoculated medium served as a negative control. Biogenic amines were quantified using UPLC-MS/MS as described below.

To assess the production of volatile organic compounds (VOCs), each culture was grown overnight in TYG broth supplemented with skim milk powder (1%, m/m; Sigma-Aldrich) and then inoculated (5%, v/v) in 2 mL of skim milk (Colruyt Group, Belgium) in UV-treated headspace vials, type N18 clear screw neck, with a maximum volume of 10 mL (Macherey-Nagel, Düren, Germany). These vials were inoculated at 30 °C for 4 days. Afterward, 20 μL of a solution of 10 ppm of 4-methyl-2-pentanol (Sigma-Aldrich) in methanol (Thermo Fischer Scientific, Carlsbad, California, United States) was added to each vial as internal standard (IS). Headspace/solid-phase microextraction coupled to gas chromatography with time-of-flight mass spectrometry (HS/SPME-GC-TOF-MS), with a Trace 1,300 gas chromatograph (Thermo Fisher Scientific) equipped with a divinylbenzene/carboxen/polydimethylsiloxane SPME fiber (50/30 μm; Supelco, Merck), and a fused silica capillary Stabilwax-MS column (Restek, Bellefonte, Pennsylvania, United States), was then applied to obtain VOC fingerprints, as described previously (Díaz-Muñoz et al., 2021). Finally, the *Lacc. paracasei* LP46 strain was selected, originating from a cheese rind (Decadt et al., 2023).

#### 2.1.2 Tetragenococcus halophilus

In total, 244 isolates of *T. halophilus* were obtained from cheese brines and rennets previously (Decadt et al., 2024b). All isolates, preserved in TYG broth supplemented with 50 g/L of NaCl (Merck), or MRS broth supplemented with either 50 g/L or 100 g/L of NaCl, all supplemented with 25% (v/v) glycerol in cryovials at −80 °C, were streaked on an agar medium consisting of MRS agar (Oxoid) supplemented with 50 g/L of NaCl (Merck), and incubated at 30 °C for 5 days. Colonies were then picked and inoculated in sterile, closed 15-mL Falcon tubes (Sarstedt, Nümbrecht, Germany) containing 8 mL of cheese medium (Neviani et al., 2009). This cheese medium was made using 120 g of Gouda cheese ripened for 4 weeks, which was cut, and then mixed with 1 L of tap water and 50 g/L of NaCl (Merck) for 5 min at medium speed using a peristaltic blender (Laboratory Blender Stomacher 400; Seward, Worthing, West Sussex, United Kingdom). The suspension was subsequently centrifuged at 1,000 × *g* for 5 min, and the supernatant was sterilized by autoclaving (121 °C, 20 min). The cultures were incubated at 30 °C for 12 days in this cheese medium. The same, not inoculated medium served as a negative control. Thereafter, the fermented cheese media were analyzed as to their free amino acid content. Based on the results, a subset of 51 fermented cheese media was analyzed for the presence of biogenic amines, and 35 of them were subsequently analyzed for VOCs. Finally, the *T. halophilus* TH63 strain was selected, originating from a brine sample (Decadt et al., 2024b).

### 2.2 Production and lyophilization of biomass of the adjunct starter culture strains

For biomass production of the strain *Lacc. paracasei* LP46, 900 mL of MRS broth was inoculated (0.5%, v/v) with an overnight culture of this strain and grown for 48 h at 30 °C. A small sample (twice 1 mL) was taken for colony enumeration by making serial tenfold dilutions, which were plated on MRS agar medium (Oxoid) supplemented with 0.1 g/L of vancomycin (Sigma-Aldrich). Furthermore, the cells were centrifuged at 5000 × *g* for 20 min at 4 °C, resuspended in 450 mL of sterile peptone saline [0.1 g/L of peptone (Oxoid) and 8.5 g/L of NaCl (Merck)] and centrifuged again applying the same conditions. All pellets were resuspended in a final volume of 180 mL of a solution containing 10% (m/v) sucrose (Sigma-Aldrich) and 10% (m/v) skim milk (Sigma-Aldrich; Berner and Viernstein, 2006; Zayed and Roos, 2004) and poured into eight Petri dishes. Then, the cells were frozen by pouring liquid nitrogen (Air Liquide, Paris, France) on these cell suspensions, whereafter the Petri dishes were kept at −80 °C for 40 min. The Petri dishes were subsequently placed in a freeze dryer (Heto Drywinner DW 6–55-1; Heto-Holten, Allerød, Denmark) for 30 h at a pressure below 2 mbar, with the cooler at −55 °C. The freeze-dried product was pulverized with a sterile spoon and put in a sterile, oven-dried glass bottle for storage at 4 °C until use.

Biomass of the strain *T. halophilus* TH63 was produced in the same way as described above, with the only difference that it was grown in MRS broth supplemented with 100 g/L of NaCl (Merck) and 30 g/L of glucose (Sigma-Aldrich) for 72 h, and then resuspended in sterile peptone saline containing 0.1 g/L of peptone (Oxoid) and 30 g/L of NaCl (Merck).

After lyophilization, 0.1 g of the biomass powder was resuspended in 1 mL of sterile saline (8.5 g/L of NaCl), and serial tenfold dilutions were plated for colony enumeration on MRS agar medium (Oxoid) supplemented with 0.1 g/L of vancomycin (Sigma-Aldrich) in the case of the *Lacc. paracasei* LP46 strain, and on MRS agar medium (Oxoid) supplemented with 100 g/L of NaCl (Merck) in the case of the *T. halophilus* TH63 strain. Using the colony count information before centrifugation and lyophilization, the survival rate was calculated using the following formula:


$$\text{Survival rate}\;(\%)=\frac{\mathrm{CFU}/g\;\text{powder}\times \text{total mass powder}}{\mathrm{CFU}/\mathrm{mL}\;\text{broth}\times \text{total volume broth}}$$

Additionally, both strains were plated on yeast extract–peptone–glucose (YPG) agar medium [5.0 g/L of yeast extract (Oxoid), 10.0 g/L of peptone (Oxoid), and 20.0 g/L of glucose (Merck)] supplemented with 0.1 g/L of chloramphenicol (Merck), and violet red-bile-glucose (VRBG) agar medium (Oxoid), to assess possible contaminations with presumptive yeasts and enterobacteria, respectively.

### 2.3 Cheese production and sampling

#### 2.3.1 Gouda cheese production batches tested

During three consecutive weeks in September 2022, six Gouda cheese production batches without and with *Lacc. paracasei* LP46 as an adjunct were performed in the pilot facility of a European dairy company. The common primary starter culture mixtures A, B, and C were used (Decadt et al., 2023). In the first week, primary starter culture mixture C was applied, first without the adjunct, resulting in batch NCL, and the next day with the adjunct, resulting in batch PCL (Table 1). The second week, the same was done for primary starter culture mixture A, resulting in batch NAL (without the adjunct) produced on the first day, and batch PAL (with the adjunct) produced on the second day. In the third week, primary starter culture mixture B was applied, the first day without adjunct (resulting in batch NBL), and the second day with the adjunct (resulting in batch PBL).

**Table 1:** Overview of the primary and adjuct starter cultures used in the eight different Gouda cheese production batches, in order of production.

Sample code	Primary starter culture	Adjunct starter culture
NCL	C	None
PCL	C	*Lacticaseibacillus paracasei* LP46
NAL	A	None
PAL	A	*Lacticaseibacillus paracasei* LP46
NBL	B	None
PBL	B	*Lacticaseibacillus paracasei* LP46
NAT	A	None
PAT	A	*Tetragenococcus halophilus* TH63

In January 2023, two additional Gouda cheese production batches were performed, this time using *T. halophilus* TH63 as an adjunct, and only using the primary starter culture mixture A, resulting in batch NAT (without the adjunct) and batch PAT (with the adjunct). Batch PAT was produced 2 days after the production of batch NAT.

#### 2.3.2 Gouda cheese production method

All pilot-scale Gouda cheese production batches resulted in six cheese wheels of about 2 kg (after brining). The cheeses were made from pasteurized milk (72 °C, 15 s) that had undergone a bactofugation and that was standardized to have a fat-to-protein ratio of 1.0. In all cases, calcium chloride and annatto were already added to the milk before the addition of the milk (150 L) to the pilot cheese vat. The milk was stirred and brought to 30.5 °C, whereafter the primary starter culture mixture (C, A, or B) was added, as well as the adjunct in the case of batches PCL, PAL, PBL, and PAT. The evening before the productions, the lyophilized adjunct starter culture strains were resuspended in 300 mL of sterile skim milk (Colruyt Group) and placed at 30 °C for 15 h (until the actual production the next day). Hereto, the exact amount of lyophilized powder needed to reach a final inoculation of 5.0 log (CFU/mL) was calculated (for these calculations, we assumed that the growth during the overnight incubation was not substantial). Animal rennet was added 40 min after the addition of the starter culture(s). Following 35 min of renneting, the curd was cut for 10 min and stirred for another 10 min. Then, the curd was left to settle for 20 min, whereafter 70 L of whey was removed and 40 L of warm water (40 °C) was added. Subsequently, sodium nitrate was added, and the curd was stirred for 80 min at a temperature of 35 °C. Thereafter, the whey was drained, and the curd was cut into six equal parts, which were put into cheese molds. The curd blocks were pressed (at 0.5 bar for 30 min, at 1 bar for 30 min, and at 2 bar for 5 min), turned in the molds, and left for about 2 h before brining in a brine of 18% (m/v) NaCl at 13 °C for 24 h. Afterward, the cheeses were ripened on wooden shelves at 13 °C. During ripening, the cheeses were regularly coated with polyvinyl acetate containing natamycin and turned.

#### 2.3.3 Sampling

Samples of the pasteurized milk in the pilot cheese vat before starter addition (referred to as M), the milk after starter addition just before renneting (M + S), the whey and curd just before draining (WD and DD, respectively), the curd just before brining (DB), and the cheese cores and rinds after 1, 8, 16, 24, and 32 weeks of ripening were collected (Table 2). An aliquot of the samples collected during the production day (M up to DB) was plated immediately after sampling in the laboratory of the cheese factory. The rest of the samples were cooled on ice for transport to the laboratory of the research group for further handling (metagenetics and meta-metabolomics).

**Table 2:** Overview of the samples taken during eight Gouda cheese production batches carried out in the pilot facility of a European dairy factory.

Sample code	Step in the process	Average time in the process (h)
M	Pasteurized milk added to the cheese vat	0.00
M + S	Cheese vat milk 30 min after starter addition	0.50
WD	Whey just before draining	3.42
DD	Curd just before draining	3.42
DB	Curd just before brining	8.43
1–32 w	Cheese core and rind	After 1, 8, 16, 24, and 32 weeks of ripening

### 2.4 Microbial enumeration

Tenfold serial dilutions of aliquots of the various milk, whey, curd, and cheese samples were plated on M17 agar medium (Oxoid) supplemented with 0.2 g/L of cycloheximide (Merck) to target presumptive lactococci and streptococci; on MRS agar medium (Oxoid) supplemented with 0.1 g/L of vancomycin (Sigma-Aldrich) and 0.2 g/L of cycloheximide (Merck), further referred to as MRSv, to target presumptive *Lactobacillaceae* (including the *Lacc. paracasei* LP46 adjunct), on YPG agar medium to target presumptive yeasts, and on VRBG agar medium to target presumptive enterobacteria, as described previously (Decadt et al., 2025a). Additionally, the samples of NAT and PAT (the two batches without and with the *T. halophilus* TH63 adjunct, respectively) were plated on MRS agar medium supplemented with 10% NaCl (Merck) and 0.2 g/L of cycloheximide (Merck), further referred to as MRSs to target presumptive HLAB (including the *T. halophilus* TH63 strain; Decadt et al., 2025a). The serial dilutions of the milk and whey samples were made with sterile saline immediately. The serial dilutions of the curd and cheese samples were based on extracts made by mixing 10 g of the sample with 90 mL of sterile saline with a peristaltic Laboratory Blender Stomacher 400 (Seward) at medium speed for 5 min. The VRBG agar media were incubated at 37 °C for 24 h, whereas the other agar media were incubated at 30 °C for 3 days, except for the MRSs agar media, which were incubated at 30 °C for 5 days. All agar media were incubated aerobically. All platings were performed in a technical triplicate.

### 2.5 Metagenetic analysis

#### 2.5.1 Cell pelleting from milk, whey, curd, cheese, and lyophilized biomass samples

The curd and cheese samples were first cut into smaller pieces of about 0.5 cm x 0.5 cm x 0.5 cm, and 20 g of sample was mixed with 120 mL of a sterile sodium citrate solution (2.0%, m/v; Sigma-Aldrich) in a Laboratory Blender Stomacher 400 (Seward) at medium speed for 5 min. These suspensions were centrifuged at 500 × *g* for 5 min to remove coarse particles. Then, the supernatants, and also the milk and whey samples, were centrifuged at 4500 × *g* for 20 min. The resulting cell pellets were resuspended in 1 mL of sorbitol buffer [1.2 M sorbitol (VWR International), 50 mM Tris-base (Calbiochem, San Diego, California, United States), pH 7.5], and centrifuged at 6000 × *g* for 10 min. Cell pellets of the lyophilized adjuncts were obtained by resuspension of 0.2 g of lyophilized powder in 2 mL of sterile saline, followed by centrifugation at 6000 × *g* for 10 min. All cell pellets were stored at −25 °C until use.

#### 2.5.2 DNA extraction

Whole-community DNA was extracted from the cell pellets, as described previously (Vermote et al., 2018). Briefly, the cell pellets were subjected to an enzymatic digestion (one incubation step with lyticase and Zymolyase and another one with lysozyme and mutanolysin), a chemical/enzymatic treatment (with sodium dodecyl sulfate and proteinase K), and a mechanical disruption (with acid-washed glass beads), followed by protein removal using a mixture of chloroform, phenol, and isoamyl alcohol, an RNase treatment, and DNA purification with a Dneasy Blood & Tissue Kit (Qiagen, Venlo, The Netherlands). The DNA purity was assessed using a NanoDrop 2000 spectrophotometer, and the DNA concentrations were measured with a Qubit 2.0 fluorometer using a Qubit dsDNA HS Assay Kit (all from Thermo Fisher Scientific).

#### 2.5.3 Bacterial metagenetics

To identify and quantify the bacterial members present in the whole-community DNA extracted from the cell pellets, amplicon-based HTS of the full-length 16S rRNA gene was performed, applying long-read PacBio sequencing technology, as described previously (Decadt et al., 2023). The long-read sequences were clustered into amplicon sequence variants (ASVs) with the DADA2 R software package (version 1.14.1), updated for long amplicon reads (Callahan et al., 2019), and with settings as described previously (Decadt et al., 2023). Taxonomy was assigned using the SILVA database (version 138; Quast et al., 2013), with the minboot parameter cut-off set at 80. Because of the elevation of *Lc*. *lactis* subsp. *cremoris* to the species level as *Lc. cremoris* (Li et al., 2021), all *Lactococcus* ASVs were aligned to the genome sequences of the type strains *Lc*. *lactis* ATCC 19435^T^ and *Lc*. *cremoris* ATCC 19257^T^ and assigned to one of these species in the case that the sequence identity was at least 99.80%. In the case the sequence identity was below 99.80%, the denomination “*Lc. lactis/cremoris*” was used.

All taxonomic data reported below are expressed as relative abundances of the total sequence reads obtained per sample.

### 2.6 Meta-metabolomic analysis

#### 2.6.1 Cheese powder and extracts

Samples of curds, cheese cores, and cheese rinds were frozen using liquid nitrogen (Air Liquide) and milled into a fine powder with a coffee grinder (De’Longhi KG49, Treviso, Italy). Aqueous extracts and ethyl acetate extracts were prepared using these powders, as described previously (Decadt et al., 2023). Briefly, 1.0 g of cheese powder was mixed with 9.0 mL of ultrapure water (Milli-Q; Merck) on a rotating wheel at 30 rpm for 30 min at room temperature, followed by centrifugation at 1,000 × g for 5 min, resulting in aqueous extracts. These extracts were stored at −25 °C until further analysis. Ethyl acetate extracts were prepared by mixing 0.5 g of cheese powder with 9.5 mL of ethyl acetate (SupraSolv® grade; Merck) and supplemented with 100 μg/L of toluene-D8 (Sigma-Aldrich) as internal standard (IS). Ethyl acetate extracts were filtered with a Millex Syringe Driven Filter Unit (Millex; Merck) and immediately used for further analysis.

#### 2.6.2 Carbohydrates

The concentrations of lactose, galactose, and glucose in the aqueous extracts were quantified with external calibration, in triplicate, by high-performance anion exchange chromatography with pulsed amperometric detection (HPAEC-PAD), using an ICS 3000 chromatograph equipped with a CarboPac PA-20 column and an ED-40 PAD detector (Thermo Fisher Scientific), as described previously (Decadt et al., 2024a). The mobile phases consisted of ultrapure water (Milli-Q, Merck) as eluent A, 100 mM NaOH (J. T. Baker, Deventer, The Netherlands) as eluent B, and 900 mM NaOH (J. T. Baker) as eluent C, with a constant flow rate of 0.4 mL/min. The following gradient was applied: 0.0 to 20.0 min, 92% A, 8% B, and 0% C; linear to 26.0 min, 80% A, 20% B, and 0% C; 26.1 to 31.0 min, 100% C; and 31.1 to 36.0 min, 92% A, 8% B, and 0% C.

#### 2.6.3 Organic acids, free amino acids, and biogenic amines

The concentrations of organic acids (citric acid, fumaric acid, gluconic acid, hippuric acid, lactic acid, malic acid, malonic acid, orotic acid, oxalic acid, pyruvic acid, succinic acid, and uric acid), free amino acids [all 20 proteinogenic amino acids as well as *γ*-aminobutyric acid (GABA), citrulline, and ornithine], and biogenic amines (agmatine, cadaverine, histamine, 2-phenylethylamine, putrescine, spermidine, spermine, tryptamine, and tyramine) in the aqueous extracts were quantified with external calibration, in triplicate, by ultra-performance liquid chromatography coupled to tandem mass spectrometry (UPLC-MS/MS), using an Acquity system equipped with an HSS T3 column (Waters, Milford, Massachusetts, United States) and a triple-quadrupole tandem mass spectrometer (Waters), as described previously (Decadt et al., 2023).

In case of the organic acids, the mobile phases consisted of an ultrapure water–methanol (Merck) mixture (98:2 [v/v]) with 0.2% (v/v) formic acid (Fluka, St. Louis, MO; eluent A) and an ultrapure water–methanol mixture (5:95) with 0.2% (vol/vol) formic acid (eluent B). The gradient elution was as follows: 0.0–1.5 min, isocratic 10% B; 1.5–3.0 min, linear from 10 to 90% B; 3.0–4.0 min, isocratic 90% B; 4.0–4.1 min, linear from 90 to 10% B; and 4.1–6.0 min, isocratic 10% B.

In case of the free amino acids, the mobile phases consisted of an ultrapure water-acetonitrile (Merck) mixture (99:1 [v/v]) with 0.05% (v/v) formic acid and 0.1% (v/v) heptafluorobutyric acid (HFBA [Sigma], eluent A) and an ultrapure water–acetonitrile mixture (1:99 [v/v]) with 0.05% formic acid and 0.1% HFBA (eluent B). The mobile phase had a constant flow rate of 0.35 mL/min with the following gradient: 0.0–1.0 min, 99% A and 1% B; 1.0–8.0 min, 30% A and 70% B; 8.1–10.0 min, 100% B; and 10.1–25.0 min, 99% A and 1% B.

In case of the biogenic amines, the mobile phase consisted of an ultrapure water–acetonitrile mixture (95:5, v/v) with 0.1% (v/v) HFBA (eluent A) and an ultrapure water–acetonitrile mixture (5:95, v/v) with 0.1% (v/v) HFBA (eluent B). The following gradient (0.23 mL/min) was applied: 0.0–5.0 min, isocratic 95% A and 5% B; 5.0–7.0 min, linear from 95 to 80% A and from 5 to 20% B; 7.0–8.0 min, linear from 80 to 30% A and from 20 to 70% B; 8.0–11.0 min, isocratic 30% A and 70% B; 11.0–12.0 min, linear from 30 to 95% A and from 70 to 5% B; 12.0–15.0 min, isocratic 95% A and 5% B.

The ratio between D-lactic acid and L-lactic acid was quantified, in duplicate, using the same UPLC-MS/MS apparatus, equipped with an Astec Chirobiotic column (Supelco; Merck), as described previously (Decadt et al., 2023). The mobile phase consisted of an isocratic flow of 15% ultrapure water with 33.3 mM ammonium acetate (VWR International) and 85% acetonitrile at a constant flow rate of 0.6 mL/min.

#### 2.6.4 Short-chain fatty acids and other volatile organic compounds

The concentrations of short-chain fatty acids, acetoin, 2,3-butanedione, aldehydes, ketones, higher alcohols, and lactones in the ethyl acetate extracts were quantified, in triplicate, by gas chromatography coupled to tandem mass spectrometry, using a Trace 1,300 gas chromatograph equipped with a TriPlus RSH autosampler (Thermo Fisher Scientific) and a DB-Wax-etr column (Thermo Fisher Scientific) coupled to a TSQ 8000 EVO triple-quadrupole mass spectrometer (Interscience, Breda, The Netherlands), as described previously (Decadt et al., 2023).

### 2.7 Statistical analysis

#### 2.7.1 Microbiological and metabolite data

All statistical analyses were performed in R (version 4.1.0; R Core Team, 2021). The metabolite data of the isolate screening were normalized by means of Z-score transformations, and visualized in heatmaps using the ComplexHeatmap package (version 2.0.0; Gu et al., 2016). Hereby, hierarchical clustering analysis was based on the Ward’s method (clustering method set to “Ward. D2”). The Euclidean distance calculation method was applied to assess the similarity between samples. Significant differences of microbial counts between the different Gouda cheese production batches were assessed using a multiple pairwise *t*-test with Bonferroni adjustment for time point series of these counts, in the case of the six production batches with and without the *Lacc. paracasei* LP46 adjunct, and by a normal pairwise *t*-test in the case of the two production batches with and without the *T. halophilus* TH63 adjunct. Microbial intra-sample diversity (alpha diversity) was assessed at the species level by calculating the inverse Simpson diversity index using the vegan package (version 2.5–7; Oksanen et al., 2020). To compare the metabolites of the eight Gouda cheese production batches during cheese ripening, a principal component analysis (PCA) of all metabolites was performed with the pcromp function in R on standardized data (z-scores; subtracting the mean and dividing by the standard deviation). Additionally, metabolite concentration differences between the eight Gouda cheese production batches were determined by a multiple pairwise *t*-test on all samples during ripening, with Bonferroni adjustment. Moreover, differences between metabolite concentrations, specifically between the Gouda cheese production batch with and without the *T. halophilus* TH63 adjunct, were determined by a normal pairwise *t*-test. In all cases, the results with a *p*-value < 0.05 were considered significant.

#### 2.7.2 Organoleptic evaluation

After 8, 16, 24, and 32 weeks of cheese ripening, triangle tests were performed with samples from the Gouda cheese production batches made with adjuncts and their corresponding negative control, i.e., for comparisons between batches NAL and PAL, between batches NBL and PBL, between batches NCL and PCL, and between batches NAT and PAT. Hereto, three cheese samples, two from the negative control and one from the cheese with the adjunct, or one from the negative control and two from the cheese with the adjunct, were tasted by 12–18 persons, who then indicated which sample they perceived as the odd one. The ratio of correct answers over all answers was then interpreted according to ISO 4120 (ISO, 2004), to indicate if there was a significant organoleptic difference (*p*-value < 0.05) between the two Gouda cheese production batches compared.

After 32 weeks of Gouda cheese ripening, seven trained cheese tasters evaluated the bitter, salty, sour, sweet, and umami taste, the buttery, fruity, and nutty notes, and the overall organoleptic quality of all six Gouda cheeses made with and without the *Lacc. paracasei* LP46 adjunct. The scores were normalized to z-scores (subtracting the mean of each descriptor for each taster and dividing by the standard deviation of each descriptor for each taster). A one-way ANOVA with post-hoc Tukey test was performed to indicate significant differences (*p*-value < 0.05) between the six Gouda cheese production batches for each organoleptic descriptor. The panel evaluated the cheese samples during one session to have the best comparison possible. Since the cheeses made with and without the *T. halophilus* TH63 adjunct were produced much later compared with the cheeses with and without the *Lacc. paracasei* LP46 adjunct, the difference in ripening time was too large to include the *T. halophilus* cheeses in the descriptive organoleptic evaluation.

Finally, Pearson correlation coefficients were calculated between the overall organoleptic quality and the metabolite concentrations, and between the overall organoleptic quality and the relative abundances of bacterial species present in the cheese cores in a relative abundance of at least 0.5% after 32 weeks of ripening.

## 3 Results

### 3.1 Screening for and selection of adjunct starter culture strains

#### 3.1.1 Lacticaseibacillus paracasei

The clearing zones appearing on the agar media tested, being an indicator of the proteolytic properties of the isolates screened, were similar for all of them. Moreover, the concentrations of free amino acids in the liquid media tested were similar for all isolates. Compared with the negative control, the concentrations of all free amino acids were, on average, lower in the inoculated media, except for *γ*-aminobutyric acid, citrulline, and valine, which had higher concentrations in the inoculated media. The ratio of amino acids in the inoculated media compared with the amino acids in the control medium was, on average, 1.09 for all amino acids for all isolates tested. The highest ratio was 2.06, which was obtained for the *Lacc. paracasei* LP46 adjunct, suggesting that this strain released free amino acids to the greatest extent.

The biogenic amine concentrations were very low for all isolates tested (Supplementary Table S1). The maximum concentrations of putrescine, spermidine, and spermine (0.04, 0.48, and 0.40 mg/L, respectively) were all obtained for one isolate (LP20). Similarly, the maximum concentrations of histamine, 2-phenylethylamine, and tyramine (0.01, 0.01, and 0.25 mg/L, respectively) were all obtained for one other isolate (LP35). The maximum concentration of cadaverine (0.01 mg/L) was found for a third isolate (LP24). Agmatine and tryptamine were not found for any isolate, nor in the control medium.

A heatmap depicting the VOCs detected showed that the VOC profiles of most isolates were comparable, whereas the VOC profiles for four isolates (2, 17, 23, and 26) deviated from those of the others (Supplementary Figure S1). Of all isolates, the selected *Lacc. paracasei* LP46 adjunct displayed the greatest peak areas for acetoin and 2,3-butanedione.

#### 3.1.2 Tetragenococcus halophilus

The concentrations of all free amino acids were higher in the inoculated cheese media than in the control cheese medium, except for citrulline. The ratio of amino acids in the inoculated cheese media compared with the control cheese medium was, on average, 34.5 for all amino acids for all isolates tested, with a maximum ratio of 53.5. The ratio of the selected *T. halophilus* TH63 adjunct was 44.7. The ratios between amino acids differed, and, hence, the isolates could be clustered in heatmaps, representing groups of isolates with a similar phenotype regarding amino acid production (data not shown). This way, the 244 isolates assessed could be reduced to a set of 51 representative isolates, containing at least one isolate from each cluster, and several isolates from the clusters with high ratios.

The concentrations of all but one biogenic amine were very low for the 51 representative isolates tested (Supplementary Table S2). Moreover, the average concentrations of cadaverine, histamine, 2-phenylethylamine, and tryptamine were lower in the inoculated cheese media than in the control cheese medium. Furthermore, agmatine, spermidine, and spermine were always below the limit of quantification. However, 16 representative isolates tested produced tyramine, with an average concentration of 29.86 mg/L. The other 35 representative isolates tested had an average tyramine concentration of 0.17 mg/L in the fermented cheese medium, whereas the control cheese medium had a concentration of 0.15 mg/L.

A heatmap depicting the VOCs for all 35 isolates tested that did not produce tyramine revealed that three isolates (66, 67, and 68) deviated from the others by large peak areas of methylesters of fatty acids (Supplementary Figure S2). The selected adjunct, *T. halophilus* TH63, had relatively high peak areas for dimethyl disulfide, ethanol, heptanal, 2-heptanone, hexanal, and o-xylene, and was mainly selected for its high amino acid concentrations. However, several other isolates had high amino acid concentrations as well; those were not selected because they produced tyramine.

### 3.2 Application of the adjunct starter culture strains

#### 3.2.1 Microbial counts

**Lyophilized adjunct starter culture biomass**. The microbial counts of the lyophilized adjunct *Lacc. paracasei* LP46 and *T. halophilus* TH63 were below the limit of detection on both VRBG agar medium and YPG agar medium. Counts of the lyophilized powder containing the *Lacc. paracasei* LP46 adjunct were 9.7 log (CFU/g) on MRSv agar medium, whereas those of the lyophilized powder containing the *T. halophilus* TH63 adjunct were 10.1 log (CFU/g) on MRSs agar medium. Consequently, the survival rate of the lyophilization process was 4% for *Lacc. paracasei* LP46 and 85% for *T. halophilus* TH63.

**Figure 1:**
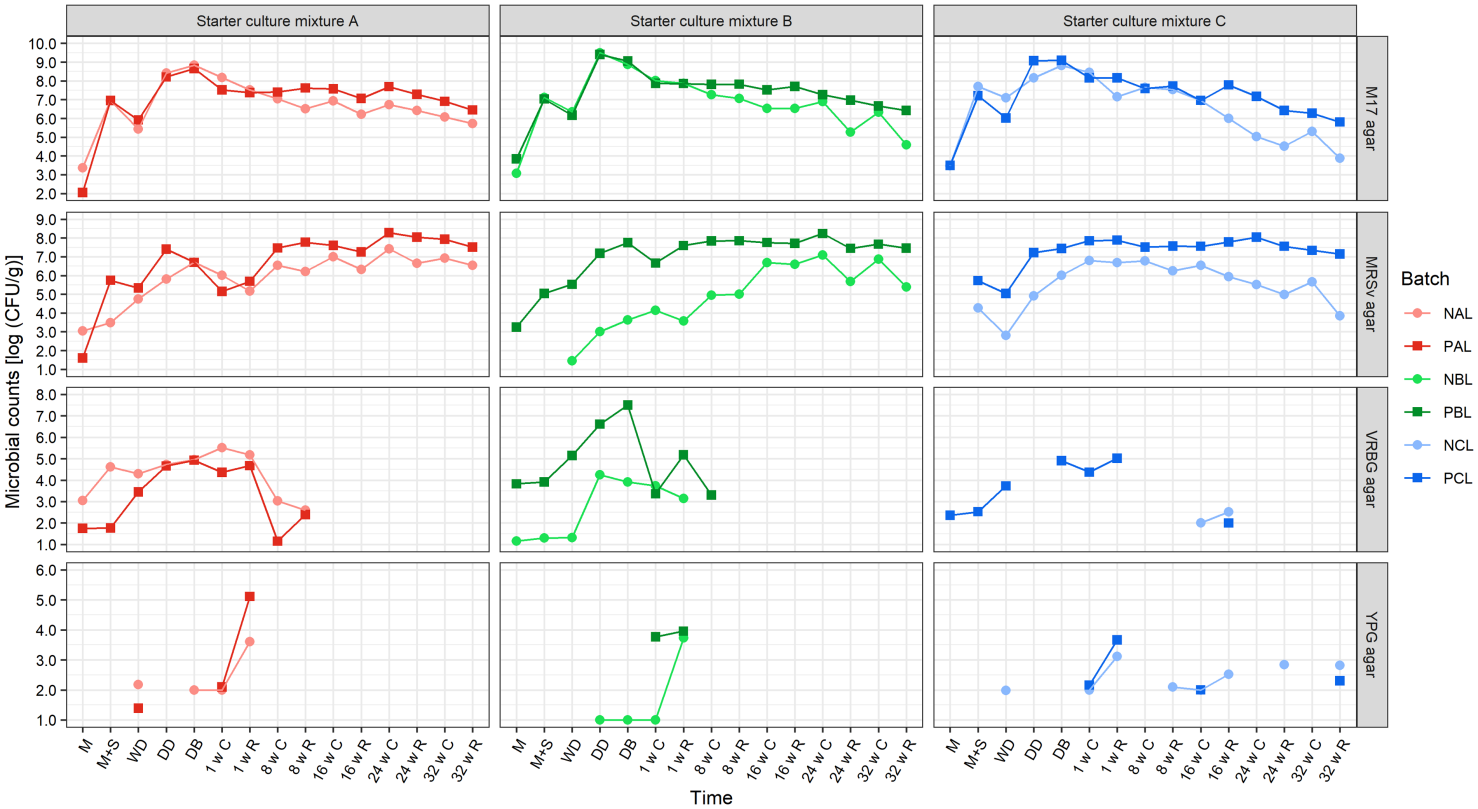
Course of the microbial counts on M17 agar medium, de Man–Rogosa–Sharpe agar medium with vancomycin (MRSv agar), violet red–bile–glucose (VRBG) agar medium, and yeast extract–peptone–glucose (YPG) agar medium, during the production day and the ripening of all Gouda cheese batches made without (light colored circles) and with (darker colored squares) the *Lacticaseibacillus paracasei* LP46 adjunct. The codes used for the different time points are as explained in Table 2. Red colors, batches made with primary starter culture mixture A; green colors, batches made with primary starter culture mixture B; blue colors, batches made with primary starter culture mixture C. Missing data points were below the limit of detection.

**Cheese production chain with the *Lacticaseibacillus paracasei* LP46 adjunct**. For the six Gouda cheese production batches with and without the *Lacc. paracasei* LP46 adjunct, the presumptive lactococcal/streptococcal counts on M17 agar medium increased from, on average, 7.2 log (CFU/g) in the milk after the primary starter culture addition to, on average, 8.9 log (CFU/g) just before brining. This increase was not only caused by growth, but also by the concentration of bacteria through whey removal. Afterward, the counts dropped with one log after 1 week of cheese ripening and slightly decreased toward an average of 6.3 log (CFU/g) in the cheese cores after 32 weeks of ripening (Figure 1). When the counts on M17 agar medium were pairwise compared between the Gouda cheese production batches with and without the *Lacc. paracasei* LP46 adjunct from the moment of addition up to 32 weeks of ripening, the productions with the adjunct added had significantly higher counts than the ones without, for each of the three primary starter culture mixtures used. However, these differences were no longer significant when comparing all six Gouda cheese production batches in a multiple pairwise *t*-test with Bonferroni adjustment; only the counts for batch NAL were significantly lower than those for batches PBL and PCL.

The presumptive *Lactobacillaceae* counts on MRSv agar medium were, on average, 2.6 and 5.5 log (CFU/g) for the Gouda cheese production batches without and with the *Lacc. paracasei* LP46 adjunct, respectively, after primary starter culture addition (Figure 1). The counts gradually increased to, on average, 6.7 and 8.2 log (CFU/g) in the cheese cores of the production batches without and with the *Lacc. paracasei* LP46 adjunct, respectively, and slightly decreased toward 32 weeks of ripening. The counts already decreased between 16 and 24 weeks of ripening in the case of batch NCL. The counts on MRSv agar medium were significantly higher for all Gouda cheese production batches with the *Lacc. paracasei* LP46 adjunct compared with all production batches without, from the moment of starter culture addition up to 32 weeks of ripening. Furthermore, batch NAL had significantly higher counts on MRSv agar medium than batch NBL, whereas no differences were found among the batches PAL, PBL, and PCL.

The presumptive enterobacterial counts on VRBG agar medium were rather high during the production of some Gouda cheese production batches [above 4.9 log (CFU/g) for NAL, PAL, PBL, and PCL just before brining], but decreased to below 3.3 log (CFU/g) from 8 weeks of ripening and upon, and below the limit of detection from 24 weeks and upon, for all production batches (Figure 1). During the production processes, the counts were significantly higher for batch PBL than for batches NBL, NCL, and PAL, and for batch NAL compared with batch NCL. The presumptive yeast counts were always below 3.0 log (CFU/g) or even below the detection limit, except after 1 week of ripening, when a maximum value of, on average, 3.9 log (CFU/g) was reached in the cheese rinds for all production batches.

**Figure 2:**
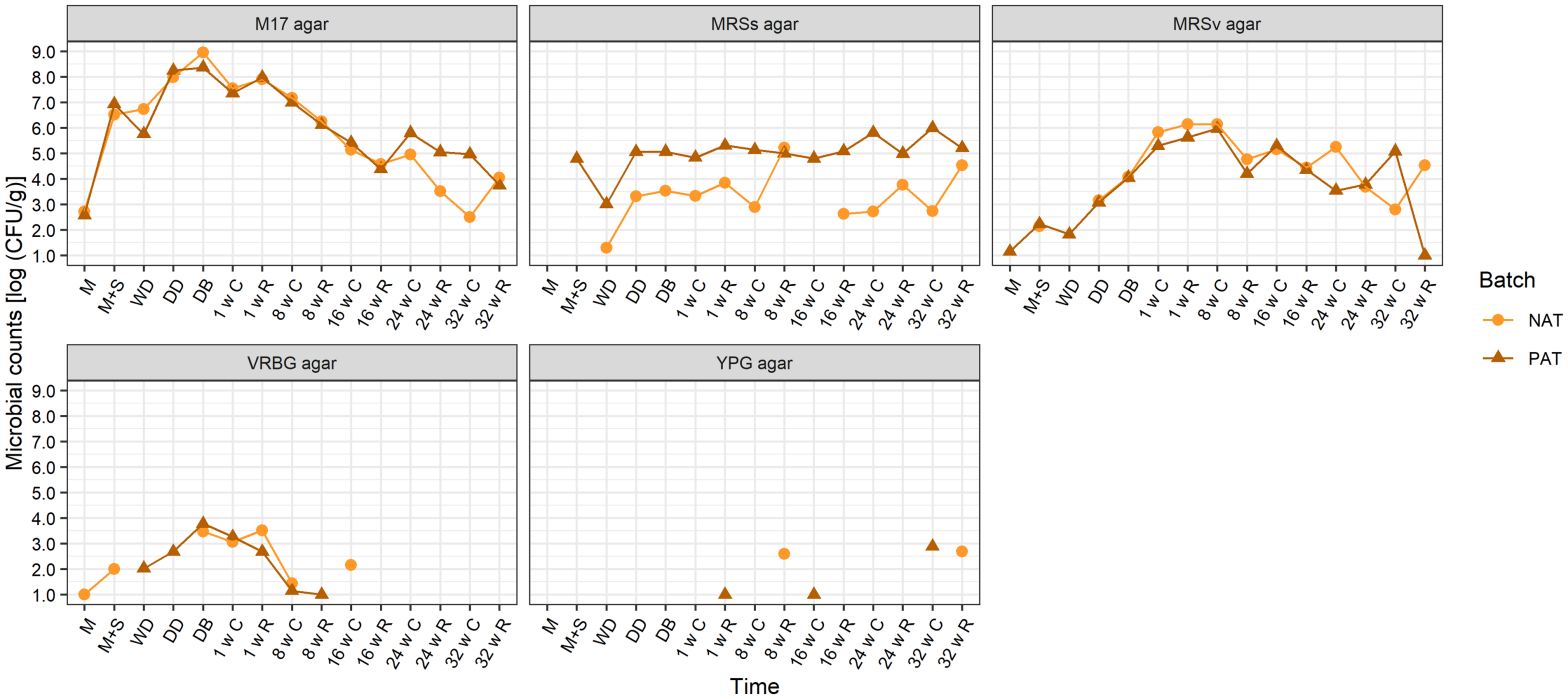
Course of the microbial counts on M17 agar medium, de Man–Rogosa–Sharpe agar medium with 10% NaCl (MRS agar), MRS agar medium with vancomycin (MRSv agar), violet red–bile–glucose (VRBG) agar medium, and yeast–peptone–glucose (YPG) agar medium, during the production day and the ripening of all Gouda cheese batches made without (orange circles) and with (brown triangles) the *Tetragenococcus halophilus* TH63 adjunct, and primary starter culture mixture A. The codes used for the different time points are as explained in Table 2.

**Cheese production chain with the *Tetragenococcus halophilus* TH63 adjunct**. For the two Gouda cheese production batches with and without the *T. halophilus* TH63 adjunct, the presumptive lactococcal/streptococcal counts reached their peak of, on average, 8.7 log (CFU/g) just before brining, and then decreased to, on average, 5.3 log (CFU/g) after 16 weeks of ripening in the cheese core, and to 2.5 and 5.0 log (CFU/g) in the cores of the cheeses of batches NAT and PAT after 32 weeks of ripening, respectively (Figure 2). The presumptive *Lactobacillaceae* counts on MRSv agar medium increased up to an average of 6.1 log (CFU/g) after 8 weeks of ripening in the cheese core, and then decreased to 2.8 and 5.1 log (CFU/g) in the cores of the cheeses of batches NAT and PAT after 32 weeks of ripening, respectively. On MRSs agar medium, the presumptive HLAB counts for batch NAT remained below 4.0 log (CFU/g) for almost all time points sampled; only the rinds of the cheeses of batch NAT had counts higher than 4.0 log (CFU/g) after 8 and 32 weeks of ripening. In contrast, the presumptive HLAB counts on MRSs agar medium for the cheeses of batch PAT increased to 4.8 log (CFU/g) after primary starter culture addition, and remained around 5.0 log (CFU/g) up to 16 weeks of ripening, to end at a maximum value of 6.0 log (CFU/g) in the cheese core after 32 weeks of ripening. From the moment the starter culture was added, the presumptive HLAB counts on MRSs agar medium for the cheeses of batch PAT were significantly higher than those for batch NAT. The presumptive enterobacterial counts on VRBG agar medium were always below 4.0 log (CFU/g) and below the limit of detection after 24 and 32 weeks of ripening. The presumptive yeast counts on YPG agar medium were mostly below the limit of detection. Apart from the significant difference in the counts on MRSs agar medium, no other significant differences were found for the microbial counts between batches NAT and PAT. Finally, as batch NAL and batch NAT were both made with primary starter culture mixture A without the addition of adjuncts, they, in fact, were biological duplicates. However, the counts on the M17, MRSv, and VRBG agar media were all significantly higher for batch NAL than batch NAT for all time points sampled.

#### 3.2.2 Metagenetics

##### 3.2.2.1 Bacterial metagenetics of whole-community DNA of Gouda cheeses made with and without the *Lacticaseibacillus paracasei* LP46 adjunct

**Figure 3:**
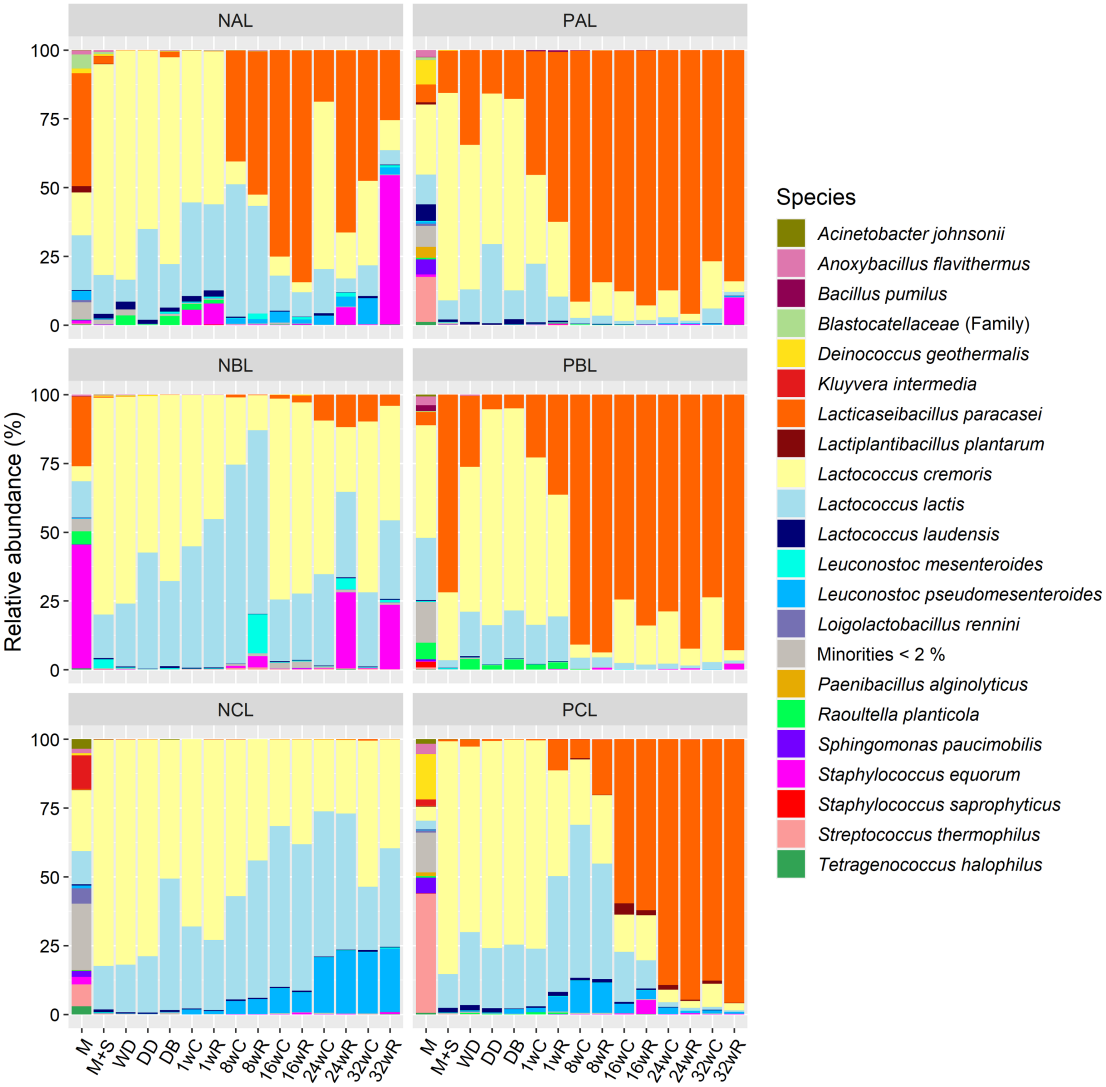
Bacterial species dynamics during the Gouda cheese production batches without (NAL, NBL, and NCL) and with (PAL, PBL, and PCL) the *Lacticaseibacillus paracasei* LP46 adjunct, made with primary starter culture mixtures A, B, and C, respectively. The sequence data are expressed as relative abundances based on amplicon sequence variants of the full-length 16S rRNA gene. The codes used for the time points are as explained in Table 2. C, core; R, rind.

All pasteurized milk samples contained a wide variety of bacterial species (Figure 3). The average alpha diversity was 5.9 (inverse Simpson; Supplementary Table S3), whereas all other samples had an alpha diversity of, on average, 2.0. After the primary starter culture addition, *Lc. cremoris* was the main species in all Gouda cheese production batches up to the brining step, with a relative abundance of, on average, 69.0%. Only after the primary starter culture addition in batch PBL, *Lacc. paracasei* was the most abundant species. For the other samples taken during the production day of batches PAL and PBL, the relative abundance of *Lacc. paracasei* was comparable with that of *Lc. lactis*, whereas it was low in the case of the other production batches (including batch PCL) during the production day. *Raoultella planticola* had a small but considerable relative abundance of, on average, 1.5 and 2.7% in production batches NAL and PBL, respectively, from the pasteurized milk up to the cheese core and rind after 1 week of ripening.

During cheese ripening, *Lacc. paracasei* became the most abundant species after 1, 8, and 16 weeks in the production batches PAL, PBL, and PCL, respectively, to remain the most abundant species until 32 weeks of ripening in the cheese cores and rinds. The average relative abundance of *Lacc. paracasei* during cheese ripening was 80.7, 74.0, and 52.8% for batches PAL, PBL, and PCL, respectively, which was significantly higher than their corresponding negative controls, which had an average relative abundance of *Lacc. paracasei* of 41.0, 4.0, and 0.1% for batches NAL, NBL, and NCL, respectively. The relative abundance of *Lacc. paracasei* was not significantly different among batches PAL, PBL, and PCL during cheese ripening. In contrast, batch NAL had a significantly higher relative abundance of *Lacc. paracasei* than batch NCL, whereas it was not significantly different than batch PCL. Indeed, *Lacc. paracasei* was the most abundant species in the case of batch NAL after 16 and 32 weeks in the cheese cores and after 8, 16, and 24 weeks in the cheese rinds.

**Figure 4:**
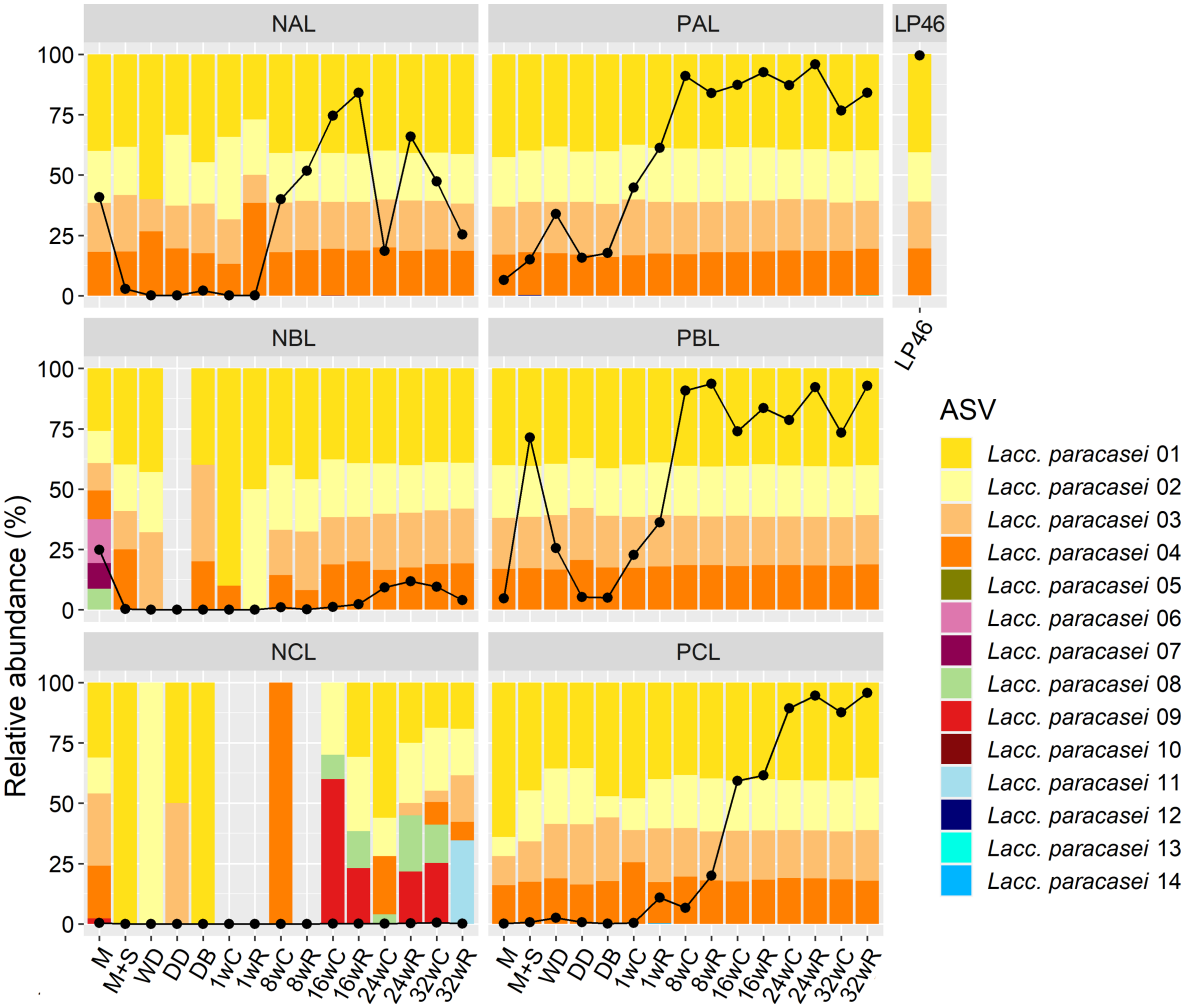
Relative abundance of the amplicon sequence variants (ASVs) for the Gouda cheese production batches without (NAL, NBL, and NCL) and with (PAL, PBL, and PCL) the *Lacticaseibacillus paracasei* LP46 adjunct, made with primary starter culture mixtures A, B, and C, respectively. The ASV composition of the adjunct is given as well. The ASVs are based on the sequencing of the full-length 16S rRNA gene of *Lacc. paracasei*. The black line indicates the relative abundance of the *Lacc. paracasei* sequence reads obtained for each sample. The codes used for the time points are as explained in Table 2. C, core; R, rind.

At the ASV level, four ASVs were found in the cheeses with the *Lacc. paracasei* LP46 adjunct, the same four, and in the same ratio, as found for the *Lacc. paracasei* LP46 adjunct when analyzed individually, namely ASV 01, 02, 03, and 04, with two copies of ASV 01 and one copy of the other ASVs (Figure 4). However, the pasteurized milk without any starter culture added used for all six Gouda cheese production batches already contained these ASVs, although in a different ratio in the case of batches NCL and PCL, which were the first two batches performed. For batch NCL, two other *Lacc. paracasei* ASVs (ASV 08 and ASV 09) were approximately equally abundant as ASVs 01–04 during cheese ripening. In the case of batch PCL, ASV 01 was more abundant in the pasteurized milk before primary starter culture addition than the *Lacc. paracasei* LP46 adjunct when analyzed individually. This suggested that the pasteurized milk probably contained several *Lacc. paracasei* strains, possibly some with the same ASVs and in the same ratio as *Lacc. paracasei* LP46, and some with perhaps only ASV 01, or ASV 01 and one other ASV. After primary starter culture addition, and especially during cheese ripening, the ratio of the *Lacc. paracasei* ASVs in batch PCL became exactly the same as found for the *Lacc. paracasei* LP46 adjunct. The other four Gouda cheese production batches (NAL, PAL, NBL, and PBL) always had the ASVs of the *Lacc. paracasei* LP46 adjunct, when *Lacc. paracasei* was abundantly present. In the case of batch NBL, three additional ASVs were abundant in the pasteurized milk, but they were no longer found during cheese production and ripening.

The relative abundance of *Lc. lactis* increased in all negative control production batches and in batch PCL during ripening. Furthermore, *Leuconostoc pseudomesenteroides* became only abundant in batch NCL, with a relative abundance of 22.3% after 32 weeks of cheese ripening. In batches NAL and PCL, *Leuc. pseudomesenteroides* had an average relative abundance over the whole ripening period of 2.9 and 4.1%, respectively. For the other production batches, the species was not abundant at all. The cores of the Gouda cheeses of batch PCL also contained some *Lactiplantibacillus plantarum*, with a maximum of 4.1% after 16 weeks of ripening. In the rinds of the Gouda cheeses of batches NAL and NBL, *Staphylococcus equorum* had a relative abundance of 13.7 and 11.1%, respectively (averages during cheese ripening), whereas this species was also found at low relative abundances in the cheese cores and rinds of all other production batches.

##### 3.2.2.2 Bacterial metagenetics of whole-community DNA of cheeses with and without the *Tetragenococcus halophilus* TH63 adjunct

**Figure 5:**
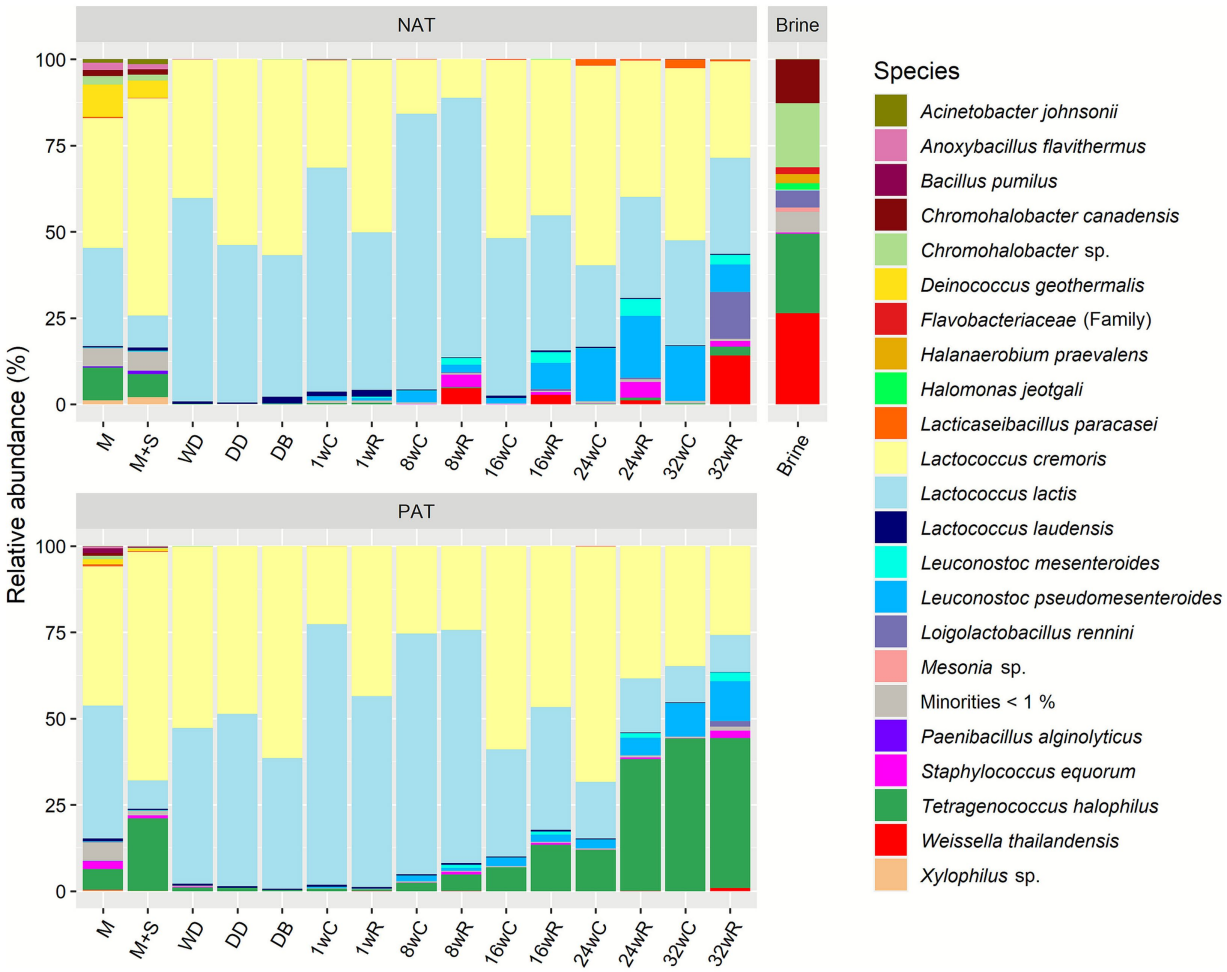
Bacterial species dynamics during the Gouda cheese production batches without (NAT) and with (PAT) the *Tetragenococcus halophilus* TH63 adjunct, made with primary starter culture mixture A. The bacterial species composition of the brine is given as well. The sequence data are expressed as relative abundances based on amplicon sequence variants of the full-length 16S rRNA gene. The codes used for the time points are as explained in Table 2. C, core; R, rind.

Similarly, for the Gouda cheese production batches with and without the *Lacc. paracasei* LP46 adjunct, the samples of the pasteurized milk used for the Gouda cheese production batch with the *T. halophilus* TH63 adjunct had a high bacterial alpha diversity, namely 4.1 and 3.2 (inverse Simpson) for batches NAT and PAT, respectively. After primary starter culture addition, the diversity decreased to values of around 2.0, to increase again after 32 weeks of cheese ripening to 2.7 and 3.0 in the cores and 4.9 and 3.6 in the rinds of batches NAT and PAT, respectively. The highest alpha diversity (5.6) was found in the brine, with *W. thailandensis*, *T. halophilus*, *Chromohalobacter canadensis,* and *Chromohalobacter* spp. as the main species (Figure 5).

After primary starter culture addition, *Lc. cremoris* and *Lc. lactis* became the most abundant species in both batches, NAT and PAT, during the production day and early cheese ripening (Figure 5). In the case of batch NAT, both species remained the most abundant ones up to 32 weeks of ripening, in the cheese cores and rinds. The third most abundant species in batch NAT was *Leuc. pseudomesenteroides*, with an average relative abundance of 7.3% during cheese ripening, whereas *W. thailandensis*, *Loigolactobacillus rennini*, *Leuc. mesenteroides*, and *S. equorum* were moderately abundant in the cheese rinds of batch NAT. *Lacticaseibacillus paracasei* was present in batch NAT, with a maximum relative abundance of 2.6% after 32 weeks in the cheese cores.

The most abundant *Lacc. paracasei* ASVs were different from those when *Lacc. paracasei* LP46 was used as an adjunct. In the case of batch PAT, the relative abundance of *T. halophilus* was high just after starter addition (20.9%) but low during further milk and cheese processing. Between 8 and 32 weeks of ripening, the relative abundance increased gradually from 2.4 to 44.2% in the cheese cores and from 4.8 to 43.4% in the cheese rinds, becoming more abundant than *Lc. cremoris* and *Lc. lactis*. The fourth most abundant species in batch PAT during cheese ripening was *Leuc. pseudomesenteroides* with an average relative abundance of 3.6%. All other species had an average relative abundance below 1.0%.

**Figure 6:**
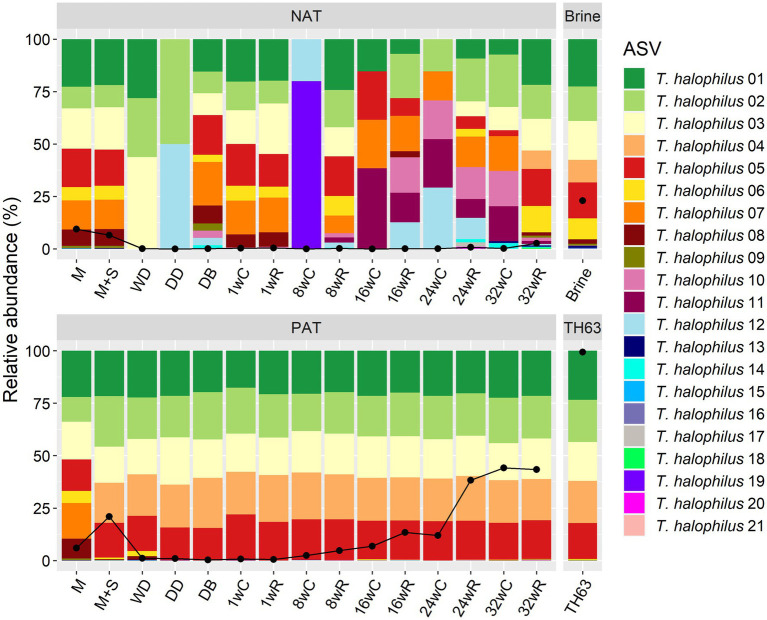
Relative abundance of the amplicon sequence variants (ASVs) for the Gouda cheese production batches without (NAT) and with (PAT) the *Tetragenococcus halophilus* TH63 adjunct, made with primary starter culture mixture A. The ASV compositions of the adjunct and the brine are given as well. The ASVs are based on the sequencing of the full-length 16S rRNA gene of *T. halophilus*. The black line indicates the relative abundance of the *T. halophilus* sequence reads obtained for each sample. C, core; R, rind.

The *T. halophilus* TH63 adjunct had five different ASVs (ASV 01–05), all equally abundant (Figure 6). All samples of the Gouda cheese production batch PAT had the same five *T. halophilus* ASVs, in the same ratio, from the moment the primary starter culture mixture was added. Four of these ASVs were also abundant in the first milk sample of both batches, NAT and PAT, together with four other ASVs (ASV 06–09). During production and ripening, several ASVs were found in the samples of batch NAT, including the same ASVs as found for *T. halophilus* TH63 when analyzed individually, although these were not the most abundant ones. The brine had six abundant ASVs (ASV 01–06), of which five were the same as those found for *T. halophilus* TH63. All ASVs found in the pasteurized milk samples were also found in the brine, except for ASV 07, which was not found in the brine.

#### 3.2.3 Meta-metabolomics

##### 3.2.3.1 Effect of the adjunct starter culture strain

**Figure 7:**
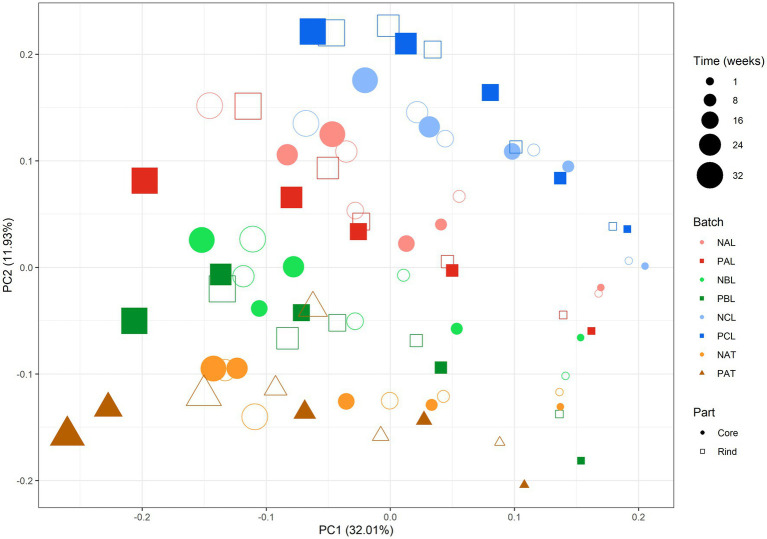
Principal component analysis based on all metabolites measured during cheese ripening across eight pilot-scale Gouda cheese production batches. The size of the shapes is proportional to the ripening time. The colors and shapes indicate the production batches: light red circles, primary starter culture mixture A without the *Lacticaseibacillus paracasei* LP46 adjunct (NAL); red squares, primary starter culture mixture A with the LP46 adjunct (PAL); light green circles, primary starter culture mixture B without the LP46 adjunct (NBL); green squares, primary starter culture mixture B with the LP46 adjunct (PBL); light blue circles, primary starter culture mixture C without the LP46 adjunct (NCL); blue squares, primary starter culture mixture C with the LP46 adjunct (PCL); orange circles, primary starter culture mixture A without the *Tetragenococcus halophilus* TH63 adjunct (NAT); brown triangles, primary starter culture mixture A with the TH63 adjunct (PAT). Filled shapes represent the cheese core samples, open shapes represent the cheese rind samples.

A PCA of all meta-metabolomic data obtained for the Gouda cheese production samples between one and 32 weeks of cheese ripening revealed that there was no consistent effect of the adjunct added (Figure 7). The main difference between the cheese samples (PC1) was related to the cheese ripening time, and this could explain 32% of the total variance. PC2 explained 12% of the total variance and was related to the production week. As batches NAL and PAL did not cluster with batches NAT and PAT, PC2 did not cluster based on the primary starter culture mixture used.

Over the whole ripening period, from one up to 32 weeks of ripening, a large number of metabolites present in the cheese cores and rinds were significantly different between two or more Gouda cheese production batches. Nevertheless, no metabolite was significantly different between all negative controls and their corresponding production batches with the *Lacc. paracasei* LP46 adjunct. However, considering that batch NAL also contained *Lacc. paracasei* (average relative abundance of 41.0% during cheese ripening), batch NAT could be considered as an alternative (and better) negative control for batch PAL, and will hence also be used as such for the metabolite comparisons below.

The concentrations of 29 metabolites differed significantly between Gouda cheese production batches NAT and PAT during the whole ripening period (without Bonferroni adjustment). When the analysis was performed for the eight Gouda cheese production batches with Bonferroni adjustment, only six metabolites differed significantly in concentration between the batches NAT and PAT, namely acetoin, 2,3-butanedione, cadaverine, dimethyl sulfone, malic acid, and pyruvic acid. Still, the higher number of significant differences might indicate that the *T. halophilus* TH63 adjunct had a larger impact on the Gouda cheeses produced than the *Lacc. paracasei* LP46 adjunct.

##### 3.2.3.2 Carbohydrates and organic acids

Lactose was the main carbohydrate present in the milk and was completely depleted after 1 week of cheese ripening in all Gouda cheese production batches performed (Supplementary Figure S3; Supplementary Table S4). Glucose and galactose had maximum concentrations of, on average, 123 and 695 mg/kg in the pasteurized milk and the cheese curd just before brining, respectively, and had low to very low concentrations during cheese ripening. L-lactic acid was the main organic acid, followed by D-lactic acid, with their highest average concentrations of 25.0 and 0.58 g/kg, respectively, in the cheese cores after 32 weeks of ripening. The highest D-lactic acid concentration was found in the Gouda cheeses of batch PCL, followed by those of batch NCL. Similarly, the Gouda cheeses of batch PBL had the highest malic acid concentrations, followed by those of batch NBL. Also, the Gouda cheeses of batch PBL had the highest concentrations of pyruvic acid. The concentrations of malic acid and pyruvic acid were significantly higher in the Gouda cheeses of batch PAT than those of batch NAT.

##### 3.2.3.3 Free amino acids and biogenic amines

The concentrations of all amino acids increased gradually during cheese ripening (Supplementary Figure S4; Supplementary Table S4). Whereas there was no effect of the *Lacc. paracasei* LP46 adjunct during the whole ripening period (see Section 3.2.3.1), the total amino acid concentrations were higher in the cheese cores after 32 weeks of ripening for all production batches with this adjunct than their corresponding negative control batches. The cores of the Gouda cheeses of batches PAL and PCL (after 32 weeks of ripening), and those of the Gouda cheeses of batch PAT (after 24 and 32 weeks of ripening) had the highest concentrations of total amino acids, as was also reflected in the PCA (Figure 7), as these samples clustered at the left-hand side of the plot. More specifically, the concentrations of 15 amino acids were significantly higher in the Gouda cheeses of batch PAT than those of batch NAT during the whole ripening. On average, the concentrations of the total amino acids were 55% higher in the Gouda cheeses of batch PAT than those of batch NAT.

The cadaverine concentrations were significantly higher in the Gouda cheeses of batches PAL, PBL, and PCL than those of the corresponding batches NAT, NBL, and NCL, respectively, with, on average, 194 and 16 mg/kg during the whole ripening period. The cadaverine concentrations were already high after 1 week of ripening in the Gouda cheeses of all production batches with the *Lacc. paracasei* LP46 adjunct, as well as in those of batch NAL, and slightly decreased during ripening. The concentrations of the other biogenic amines remained very low. However, the cadaverine concentrations of the Gouda cheeses of batch PAT were significantly lower than those of the Gouda cheeses of batches NAL, PAL, and PBL, and they were significantly higher than those of the batches NAT, NBL, and NCL.

##### 3.2.3.4 Short-chain fatty acids and volatile organic compounds

The concentrations of all short-chain fatty acids displayed an increasing trend during cheese ripening (Supplementary Figure S5; Supplementary Table S4). Acetic acid was the short-chain fatty acid with the highest concentration, followed by butyric acid, with average concentrations in the cheese cores of 1,725 and 102 mg/kg, respectively, after 32 weeks of ripening. Whereas the concentrations of dimethyl sulfone and 2-nonanone remained stable, a decreasing trend was found for other VOCs, such as acetoin and dimethylsulfoxide. The VOCs 1-butanol, ethyl dodecanoate, hexanal, 1-hexanol, methyl butyrate, 1-nonanol, 2,4-pentanediol, 2-pentanol, and *δ*-undecalactone were only found in some Gouda cheese batches.

The acetoin concentrations were significantly higher in the Gouda cheeses of batches PAL and PBL than in the corresponding ones of batches NAT and NBL. The greatest difference occurred for the Gouda cheeses of the NBL/PBL batch pair, with average acetoin concentrations of 141 and 253 mg/kg during ripening, respectively. The acetoin concentrations were also higher in batch PCL than those in batch NCL, but that was only significant in a pairwise *t*-test without Bonferroni adjustment. Furthermore, there was a trend of higher concentrations of isobutyric acid, 2-methyl-butanoic acid, and 3-methyl-butanoic acid associated with the *Lacc. paracasei* LP46 adjunct, but this was only significant between 16 and 32 weeks for the NCL/PCL pair in a pairwise *t*-test without Bonferroni adjustment.

The Gouda cheeses of batch PAT had significantly higher concentrations of acetoin and the related compounds 2,3-butanedione and tetramethylpyrazine than those of batch NAT.

#### 3.2.4 Organoleptic evaluation

Triangle tests using cheeses of production batches with the same primary starter culture mixture, but with and without adjunct, showed only twice a significant organoleptic difference, namely for the Gouda cheeses of batches NAL and PAL after 16 weeks of ripening, and for those of batches NCL and PCL after 24 weeks of ripening (Supplementary Table S5). Hence, the Gouda cheeses made with adjuncts were, in general, not tasted differently from the cheeses made without adjuncts.

When organoleptically evaluating the six Gouda cheeses with and without the *Lacc. paracasei* LP46 adjunct, it turned out that cheeses from batches NAL and NBL were significantly more fruity than those of batch NCL (Supplementary Figure S6). The category nutty flavor was scored significantly higher for Gouda cheeses of batch NAL than those of the batches NCL and PCL. Finally, Gouda cheeses of batches NAL, PAL, PBL, and PCL scored all significantly higher in overall quality than those of batch NCL.

Of all metabolites, only cadaverine and 3-methyl-butanoic acid had a significant positive correlation with the overall quality of the Gouda cheeses produced, whereas no metabolites had a significant negative correlation with the overall quality. Of the species found at a relative abundance higher than 0.5%, only *Lacc. paracasei* had a significant positive correlation with the overall quality of the Gouda cheeses produced.

## 4 Discussion

In general, Gouda cheese production is steered by the use of primary starter cultures, but the organoleptic properties of the final cheeses are influenced by the growth of NSLAB during cheese ripening, as species of the latter are part of the house microbiota and present in the pasteurized milk, in the brine, and on processing surfaces (Bokulich and Mills, 2013; Decadt and De Vuyst, 2023; Decadt et al., 2023, 2024a; Decadt et al., 2024b). As these NSLAB species may be of positive impact, they are often used as adjuncts to accelerate cheese ripening and, hence, have an impact on the cheese flavor profile (Gobbetti et al., 2015). Whereas the culture-dependent and metagenetics data demonstrated a successful inoculation of the adjuncts applied in the present study, the metabolomics data suggested a limited impact on the flavor of the Gouda cheeses produced in the case of the *Lacc. paracasei* LP46 adjunct, and a moderate impact in the case of the *T. halophilus* TH63 adjunct. However, for both adjuncts, the organoleptic evaluation could not demonstrate any significant perceived differences between the Gouda cheeses made with and without these adjuncts.

A first advantage for enhanced cheese flavor formation during ripening is the release of amino acids upon proteolysis. In the current study, the total amino acid concentrations were only higher after 32 weeks of ripening in all Gouda cheeses made with the *Lacc. paracasei* LP46 adjunct than their negative control. Usually, the addition of *Lacc. paracasei* increases the proteolysis degree (Saiki et al., 2018; Van Hoorde et al., 2010), although this effect is not always significant (Bielecka and Cichosz, 2017). Hence, as higher amino acid concentrations are desirable from an early stage of the cheese ripening stage onward, the *Lacc. paracasei* LP46 adjunct did not seem to be the best choice, as an increased proteolysis was moderate at best. In contrast, significantly higher concentrations of total amino acids were obtained with the application of the *T. halophilus* TH63 adjunct than its negative control throughout the whole ripening period. As no reports exist on the use of *T. halophilus* as a cheese adjunct, it can only be compared with its application in other fermented foods. Whereas *T. halophilus* is strongly correlated with the production of several amino acids in the case of soy sauce fermentation processes (Zhang et al., 2020), this correlation is moderate in the case of shrimp sauce fermentation processes (Udomsil et al., 2011), and absent in the case of fish sauce fermentation processes (Kim et al., 2019). Focusing on dairy isolates, a previous study has found that the proteolytic properties of *T. halophilus* strains in skim milk fermentation processes are superior to all other HLAB species isolated from cheeses, including *Lacc. rhamnosus* and *Lacp. plantarum* (Morales et al., 2011). Additionally, *T. halophilus* is able to hydrolyze casein according to phenotypical tests (Morales et al., 2011; Unno et al., 2020). Although another study has not found production of caseinolytic proteinases (Rodríguez et al., 2022), those enzymes have been detected in metagenome-assembled genomes of *T. halophilus* (Decadt et al., 2025b). Hence, the *T. halophilus* TH63 adjunct showed promising results, but more pilot-scale cheese production batches are needed to confirm its impact on proteolysis in cheese and to investigate its industrial applicability.

In the case of the *Lacc. paracasei* LP46 adjunct, the significantly high concentrations of cadaverine in the Gouda cheeses made with this adjunct were not in line with the data of the isolate screening experiments, as no single isolate produced cadaverine in the medium tested. However, there is evidence that this strain contained a decarboxylase gene, involved in the production of biogenic amines. Indeed, the retrieved metagenome-assembled genome of *Lacc. paracasei* obtained through metagenomic sequencing and analysis of several Gouda cheeses, including B7, from which isolate LP46 was obtained, contained an inducible ornithine decarboxylase (*odcI*) gene (Decadt et al., 2025b). In addition, several predicted genes that showed high sequence identity (> 99%) with an *odcI* gene of *Lacc. paracasei* were present in the metagenome of cheese B7 only (Decadt et al., 2025b). Although the encoded ornithine decarboxylase catalyzes the decarboxylation of ornithine to putrescine, it has a minor activity on lysine to convert it to cadaverine (Young et al., 2018). Also, it is known that some LAB genes annotated as *odcI* are actually lysine decarboxylases (*ldc*; Berthoud et al., 2022; Romano et al., 2013). Moreover, the concentrations of cadaverine are three times higher than those of putrescine in the case of cheeses made with a *Paucilactobacillus wasatchensis* adjunct that contains two *odcI* genes (Berthoud et al., 2022). It is likely that the *Lacc. paracasei* LP46 adjunct only contained a functional *ldc*, given the very low putrescine concentrations in all Gouda cheeses produced during the present study. Already after 1 week of ripening, the cadaverine concentrations were highest, whereas in general, biogenic amine concentrations increase during cheese ripening (Komprda et al., 2007; Linares et al., 2012). The unusual regulation of cadaverine in the *Lacc. paracasei* LP46 adjunct might be similar to that of *Serratia proteamaculans* in beef, with maximum gene expression of *ldc* during the exponential growth phase, and maximum cadaverine production during the early stationary phase (De Filippis et al., 2013). Additionally, the pH range for cadaverine production by the Ldc protein of *P. wasatchensis* is rather narrow, with an optimal pH of 5.5 (Berthoud et al., 2022). If the cadaverine production by the *Lacc. paracasei* LP46 adjunct would have a similar pH optimum; it might have been reached only during brining, when the pH is at its lowest level in the Gouda cheese production chain (Decadt et al., 2024a). Additionally, brining causes bacterial stress, which can further increase the biogenic amine production (Barbieri et al., 2019). Due to the adverse health effects of biogenic amines, a cadaverine-producing adjunct should, in fact, never be considered. However, cadaverine was one of the few compounds that showed a significant positive correlation with the overall organoleptic quality, and because of the high correlation between cadaverine and the *Lacc. paracasei* LP46 adjunct, the latter species was also positively correlated with the overall organoleptic quality. Although high concentrations (> 1,500 mg/kg) of cadaverine are negatively correlated with overall organoleptic quality in Gouda cheeses (Decadt et al., 2023), cadaverine has been suggested as a contributor to the desired spicy trigeminal sensation (burning taste) of Parmesan cheese (Hillmann and Hofmann, 2016). Its correlation with the organoleptic quality of cheeses might be positive at low concentrations, and negative at high concentrations. Concerning its toxicity, the no-observed-adverse-effect level of cadaverine is 180 mg/kg body weight/day for rats (Til et al., 1997), but seven times lower in the case of human cell lines (del Rio et al., 2019). Still, concentrations of 300 mg/kg cheese are supposed to be safe for normal to high cheese consumption. Hence, if cadaverine really contributes to the cheese’s overall organoleptic quality, an adjunct producing a controlled, low concentration would not necessarily be disqualified.

As the metagenetics data, and more specifically the ASVs and their ratios, indicated a considerable relative abundance of the *Lacc. paracasei* LP46 adjunct in the processed milk and cheese samples of batch NAL, made without adjunct, the similar cadaverine concentration present was very likely produced by the *Lacc. paracasei* LP46 adjunct added, indicating a cross-contamination between production batches. Although the cheese vats were thoroughly cleaned and disinfected after each use, a more thorough cleaning procedure would be needed to ensure the absence of *Lacc. paracasei* LP46 in the cheese vat after a Gouda cheese production with this adjunct. However, this might be challenging, since cleaned and disinfected cheese vats usually contain bacterial counts of around 3.0 log (CFU/cm^2^; Calasso et al., 2016). Based on the ASV profiles, batch NBL probably also got cross-contaminated with *Lacc. paracasei* LP46. The absence of *Lacc. paracasei* LP46 in batch NCL confirmed the hypothesis of a cross-contamination between production batches, since batch NCL was the very first batch produced of the whole series of pilot-scale batches performed. Furthermore, high microbial counts for presumptive enterobacteria and considerable relative abundances of *R. planticola* were present in batches NAL and PBL. The latter coliform species is present at dairy farms and is associated with mastitis (Massé et al., 2020; Zadoks et al., 2011). Moreover, this bacterial species has been found in pasteurized milk, in which it can grow at low temperatures (Masiello et al., 2016). Furthermore, it has the ability to form biofilms (Massé et al., 2020). The increase of presumptive enterobacterial counts for each new Gouda cheese production batch with and without the *Lacc. paracasei* LP46 adjunct suggested biofilm formation in the pilot cheese vat, which most likely got contaminated with *R. planticola* by the pasteurized milk used for the first or second production batches (NCL or PCL). Likely, the biofilm also played a role in the inoculation of *Lacc. paracasei* LP46 in batches NAL and NBL. Compared with large, industrial cheese vats, pilot cheese vats might be more sensitive to biofilm formation, due to the manual cleaning and disinfection and the higher surface/volume ratio. This is a factor to take into account for future pilot-scale experiments, since the experimental setup of the current study was compromised by the incidental contamination of *Lacc. paracasei* LP46 in the negative controls, especially in batch NAL. However, this phenomenon demonstrated the competitiveness of *Lacc. paracasei* LP46. Finally, the low presumptive enterobacterial counts during the production of batches NAT and PAT indicated that the (presumed) biofilm might have lost its viability after some months of not using the pilot cheese vat. This was also confirmed by a very low relative abundance of *Lacc. paracasei* LP46 in batches NAT and PAT, and the fact that the most abundant *Lacc. paracasei* ASVs were different from those of strain LP46.

*Lacticaseibacillus paracasei* LP46 produced high acetoin concentrations, as revealed by both the screening experiments and the Gouda cheeses produced with this adjunct compared with those made without this adjunct. An increase in acetoin concentrations by *Lacc. paracasei* has been reported previously (Bancalari et al., 2020). Furthermore, Gouda cheeses are made with a *Lacc. paracasei* adjuncts are mainly characterized by higher concentrations of compounds derived from proteolysis, such as isobutyric acid, 2-methyl-butanoic acid, and 3-methyl-butanoic acid (Van Hoorde et al., 2010). The concentrations of the latter three compounds were indeed higher in the cheeses with the *Lacc. paracasei* LP46 adjunct, although only from 16 weeks of ripening on, and not always significant. The higher concentrations of acetoin and 2,3-butanedione in the Gouda cheeses of batch PAT than those of batch NAT could possibly be linked with the higher concentrations of pyruvic acid in batch PAT. Indeed, *T. halophilus* produces high pyruvate concentrations (Rodríguez et al., 2022). In contrast, the high concentrations of hexanal and 2-heptanone made by the *T. halophilus* TH63 adjunct during the screening experiments were not confirmed in the pilot cheeses made. However, during screening, these compounds were only quantified relative to those of the other isolates, and their absolute concentrations might still be low for *T. halophilus* TH63. Similarly, strains of *T. halophilus* contribute little to the volatile profiles of fermented fish sauces (Udomsil et al., 2011).

The screening and selection of the appropriate adjunct strain performed during the present study slightly differed for *Lacc. paracasei* compared with *T. halophilus*. In the case of the *Lacc. paracasei* isolates, the TYG medium used for the screening of their proteolytic properties probably included too high amino acid concentrations to allow reliable measurements. Additionally, the optimized medium to test the potential of biogenic amine production (Bover-Cid and Holzapfel, 1999) failed to detect the cadaverine production by *Lacc. paracasei* LP46. In contrast, the cheese medium applied for the screening of the *T. halophilus* isolates demonstrated the proteolytic properties of *T. halophilus* TH63, and allowed the detection of tyramine production by some of the isolates. Hence, this cheese medium might have been the better option for all screening experiments, underlining that test conditions should always mimic as much as possible the conditions of the real application. Some *T. halophilus* isolates were associated with high levels of methylesters, which might have been formed from short-chain fatty acids and the methanol that was part of the internal standard during the GC analysis. Hence, these isolates might have lipolytic properties, which could be desired to some extent. Therefore, their application could be considered in future pilot experiments. Additionally, an adjunct composed of multiple strains of *Lacc. paracasei* and/or *T. halophilus* could be considered, as several strains might have slightly different properties that may yield wider and more desirable flavor profiles. The combination of both species would mimic the succession of early (*Lacc. paracasei*) and late (*T. halophilus*) NSLAB species growth, which occurs in the Gouda cheeses produced without adjunct (Decadt et al., 2023, 2024a).

Another reason for the rather limited impact of the adjuncts tested (especially *Lacc. paracasei* LP46) could be that they did not add sufficient metabolic activities, in addition to the pathways of the primary starter cultures. Also, they might have partially replaced strains of the primary starter culture in the overall microbial ecosystem, instead of complementing them. Indeed, a trend occurred that showed the presence of *Lacc. paracasei* was accompanied by a lower relative abundance of leuconostocs, as has been described before (Porcellato et al., 2013). Hence, *Leuc. pseudomesenteroides* was only abundant in batch NCL. Also, cheeses with primary starter culture mixture B are characterized by a lack of leuconostocs (Decadt et al., 2023), explaining their lack in batch NBL. Furthermore, the presence of contaminating *Lacc. paracasei* LP46 in the cheese vat from the previous production batch might have repressed the *Leuconostoc* strains of starter culture mixture A during batch NAL. Similarly, fewer leuconostocs and also fewer NSLAB occurred in the Gouda cheeses made with the *T. halophilus* TH63 adjunct compared to its negative control. Possibly, *T. halophilus* TH63 might be better regarding the suppression of NSLAB than *Lacc. paracasei* LP46, as batch PCL contained some *Lacp. plantarum*. Although *Lacc. paracasei* LP46 became the most abundant species in all Gouda cheeses made with this adjunct; its outgrowth happened at a different speed, being fastest in batch PAL and slowest in batch PCL, depending on the primary starter culture mixture used. This suggested that the primary starter culture mixtures differed in their competitiveness toward *Lacc. paracasei* LP46. Hence, it is of utmost importance to test adjuncts with all primary starter culture mixtures used in a rotation system for cheese production, as the use of different primary starter culture mixtures does influence the final cheese quality (Decadt et al., 2023).

Ideally, multiple, individual isolates would have been assessed as adjuncts in a pre-pilot cheese production with the primary starter culture mixtures in use, to investigate their interactions and net contributions to the final cheese flavor. For example, a microcheese system allows a high-throughput cheese-like production (Bachmann et al., 2009). Alternatively, cheese ripening could be modeled virtually (a digital twin of real cheese) by combining (meta)genomics data of the primary starter culture mixtures and the NSLAB isolates, supplemented with some experimental data (Jamshidi et al., 2023; Lecomte et al., 2023). Both approaches could be considered in future research strategies.

## 5 Conclusion

Two adjuncts, namely, the inoculated strains *Lacc. paracasei* LP46 and *T. halophilus* TH63 became abundant in the Gouda cheeses produced during the present study. However, both adjuncts did not affected the organoleptic quality of the cheeses made. Although there was an indication of increased proteolysis after 32 weeks of cheese ripening, the only significant effect of the *Lacc. paracasei* LP46 adjunct had a higher concentration of acetoin and cadaverine. The cadaverine concentration was correlated with a positive organoleptic score and remained far below toxic levels. Hence, flavor-wise, an adjunct producing minimal amounts of cadaverine could be considered. Furthermore, the *Lacc. paracasei* LP46 adjunct became abundant at a different speed for each of the three primary starter culture mixtures applied, and it suppressed the growth of *Leuc. pseudomesenteroides* is present in these primary starters. Cross-contamination of the pilot cheese vat resulted in high enterobacterial counts during the cheese production and the occurrence of *Lacc. paracasei* LP46 adjunct in two negative control cheeses. The *T. halophilus* TH63 adjunct increased the total amino acid concentrations in the cheeses by 55% compared to its negative control, and its application also resulted in higher acetoin and 2,3-butanedione concentrations, indicating that this adjunct could be a promising cheese adjunct starter culture.

## Data Availability

The datasets presented in this study can be found in online repositories. The names of the repository/repositories and accession number(s) can be found at: https://www.ebi.ac.uk/ena, PRJEB81476.
