# Supplementary material for: Application of Lacticaseibacillus paracasei and Tetragenococcus halophilus as adjunct starter cultures in Gouda cheese production
Source: Front Microbiol. 2026 Jan 16;16:1719725. doi: 10.3389/fmicb.2025.1719725 (PMC12855489; doi:10.3389/fmicb.2025.1719725)
Supplement: Supplementary file 1 [file Data_Sheet_1.pdf]

Supplementary Material

1 Supplementary Tables

Table S1. Assessment of the biogenic amine production potential of 49 *Lacticaseibacillus paracasei* isolates, including the chosen *Lacc. paracasei* LP46 adjunct. Agmatine was below the limit of quantification.

**Biogenic amine**	**Concentration (mg/L)**			
	**Control medium**	**Average for all isolates**	**Maximum for all isolates**	**Adjunct LP46**
Cadaverine	0.001	0.003	0.015	0.003
Histamine	0.064	0.006	0.011	0.006
2-Phenylethylamine	0.008	0.006	0.009	0.005
Putrescine	0.002	0.007	0.039	0.007
Spermine	0.019	0.019	0.391	0.010
Spermidine	0.014	0.042	0.477	0.039
Tyramine	0.067	0.128	0.248	0.122

Table S2. Assesment of the biogenic amine production potential of 51 *Tetragenococcus halophilus* isolates, including the selected *T. halophilus* TH63 adjunct. Agmatine, spermine, and spermidine were below the limit of quantification.

**Biogenic amine**	**Concentration (mg/L)**			
	**Control cheese medium**	**Average for all isolates**	**Maximum for all isolates**	**Adjunct TH63**
Cadaverine	0.460	0.280	0.706	0.152
Histamine	0.052	0.033	0.090	0.015
2-Phenylethylamine	0.001	0.001	0.003	0.000
Putrescine	0.120	0.142	0.367	0.118
Tryptamine	0.005	0.002	0.009	0.001
Tyramine	0.146	9.485	41.016	0.117

Table S3. Alpha diversity (inverse Simpson) during the Gouda cheese production batches without (NAL, NBL, and NCL) and with (PAL, PBL, and PCL) the *Lacticaseibacillus paracasei* LP46 adjunct, made with primary starter culture mixtures A, B, and C, respectively, and during the Gouda cheese production batches without (NAT) and with (PAT) the *Tetragenococcus halophilus* TH63 adjunct, made with primary starter culture mixture A. Time points are explained in Table 2. C, core; R, rind.

Time point	Cheese batch							
	NAL	PAL	NBL	PBL	NCL	PCL	NAT	PAT
M	4.22	8.00	3.45	4.40	10.71	4.44	4.13	3.16
M+S	1.65	1.67	1.55	1.73	1.43	1.36	2.42	2.04
WD	1.42	2.45	1.62	2.69	1.43	1.90	1.97	2.08
DD	1.89	2.46	1.99	1.56	1.51	1.62	2.01	2.06
DB	1.69	1.90	1.81	1.74	2.07	1.64	2.04	1.92
1wC	2.36	2.84	2.01	2.26	1.81	1.62	1.93	1.61
1wR	2.42	2.16	2.02	2.81	1.67	2.93	2.18	2.02
8wC	2.48	1.20	1.72	1.21	2.14	2.60	1.50	1.82
8wR	2.35	1.38	2.07	1.14	2.24	3.44	1.72	1.93
16wC	1.71	1.29	1.72	1.64	2.23	2.44	2.10	2.23
16wR	1.39	1.16	1.85	1.38	2.30	2.34	2.74	2.75
24wC	2.31	1.29	2.32	1.52	2.58	1.25	2.42	1.97
24wR	2.11	1.09	4.10	1.17	2.72	1.11	3.58	3.12
32wC	2.92	1.60	2.14	1.67	2.60	1.29	2.73	2.97
32wR	2.67	1.39	3.21	1.16	2.97	1.09	4.94	3.55

Table S4. Concentrations of all quantified metabolites during the six Gouda cheese production batches without and with the *Lacticaseibacillus paracasei* LP46 adjunct, and during the two Gouda cheese production batches without and with the *Tetragenococcus halophilus* TH63 adjunct. The codes of the production batches are as in the legend of Figure 7. The codes used for the time points are as explained in Table 1. C, core; R, rind. n.d.; not detected. See the supplemented Excel sheet.

Table S5. Scores of the organoleptic evaluation of the Gouda cheeses produced with and without the *Lacticaseibacillus paracasei* LP46 and *Tetragenococcus halophilus* TH63 adjuncts, as performed through triangle tests. These scores are expressed as the correct answers (recognising the odd sample correctly) over all answers. Scores indicating a significant organoleptic difference between two cheese batches compared (*p*-value < 0.05) are indicated in bold. N, without adjunct; P, with adjunct; L, *Lacc. paracasei* LP46 adjunct; T, *T. halophilus* TH63 adjunct; A, B, and C refer to the primary starter culture mixtures applied.

**Gouda cheese production**	**Weeks of ripening**			
**Batches compared**	**8**	**16**	**24**	**32**
NAL/PAL	8/17	**10/18**	8/18	4/18
NBL/PBL	2/17	7/18	6/17	3/15
NCL/PCL	6/12	7/16	**9/16**	4/17
NAT/PAT	3/15	7/16	7/17	8/18

2 Supplementary figures

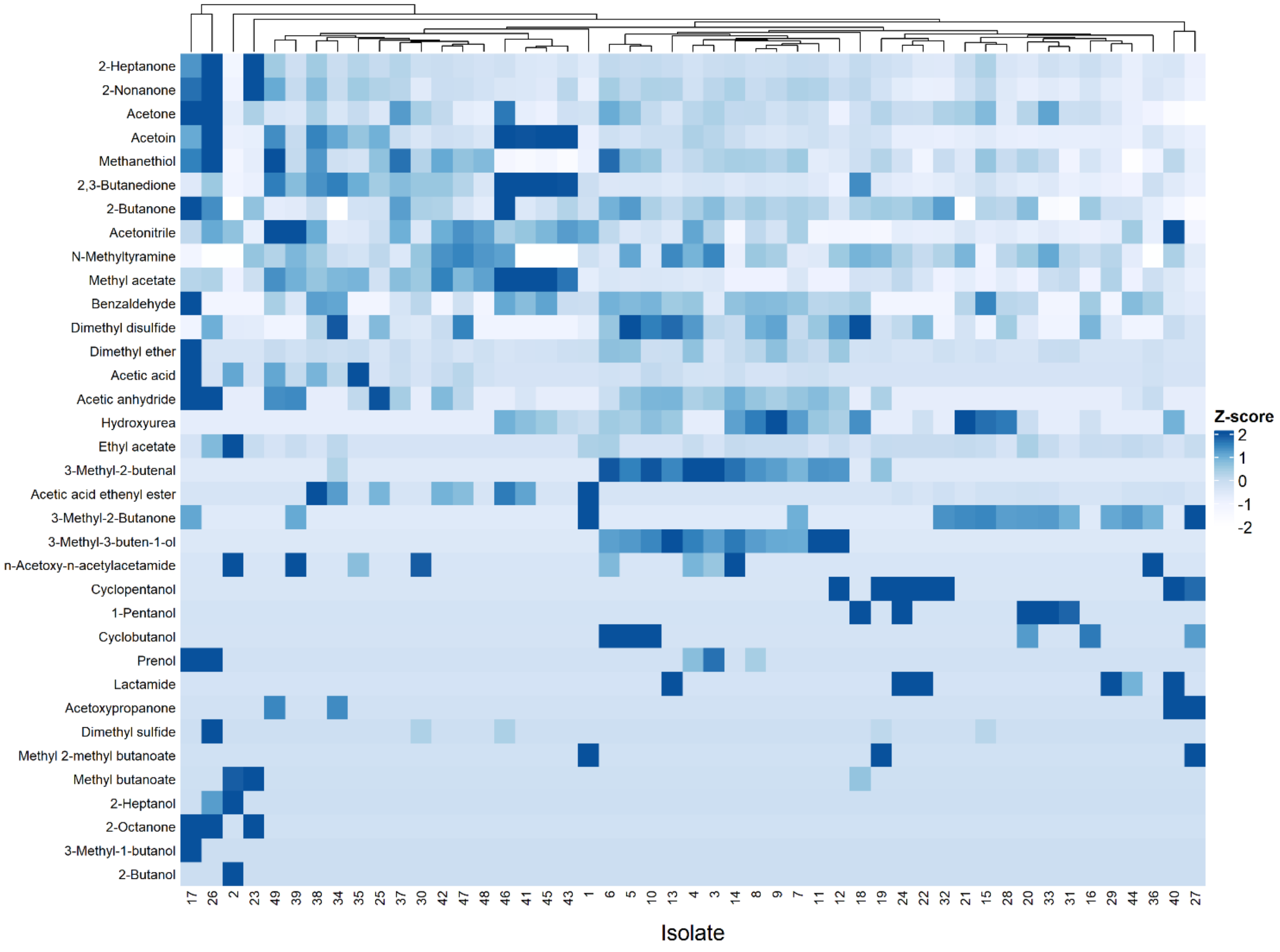

Figure S1. Heatmap of the volatile organic compounds produced in skim milk by 49 *Lacticaseibacillus paracasei* isolates. Peak areas are expressed as z-scores; high values are depicted in blue, low values are depicted in white.

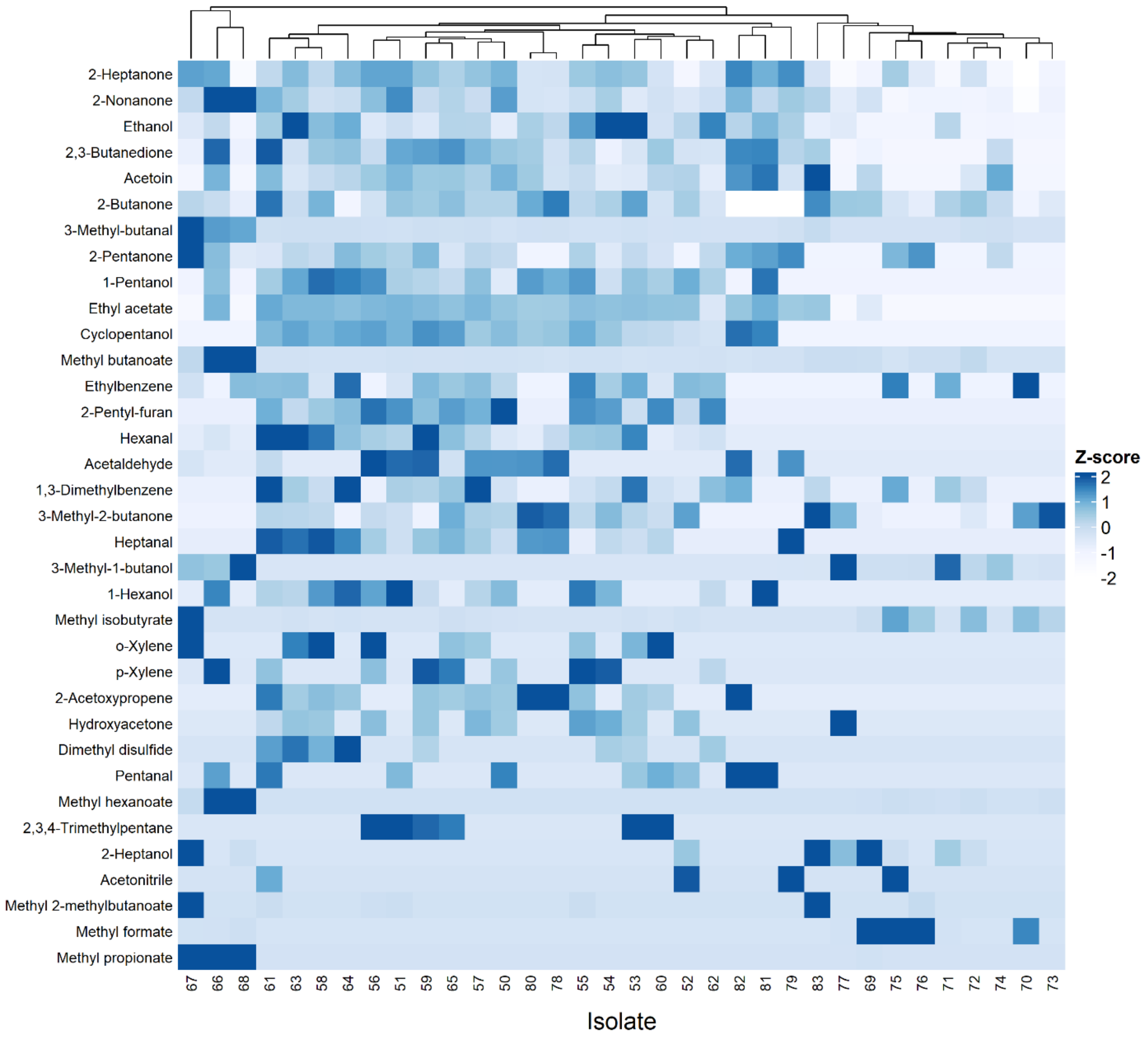

Figure S2. Heatmap of the volatile organic compounds produced in cheese medium by 35 *Tetragenococcus halophilus* isolates that did not produce tyramine. The peak areas are expressed as z-scores; high values are depicted in blue, low values are depicted in white.

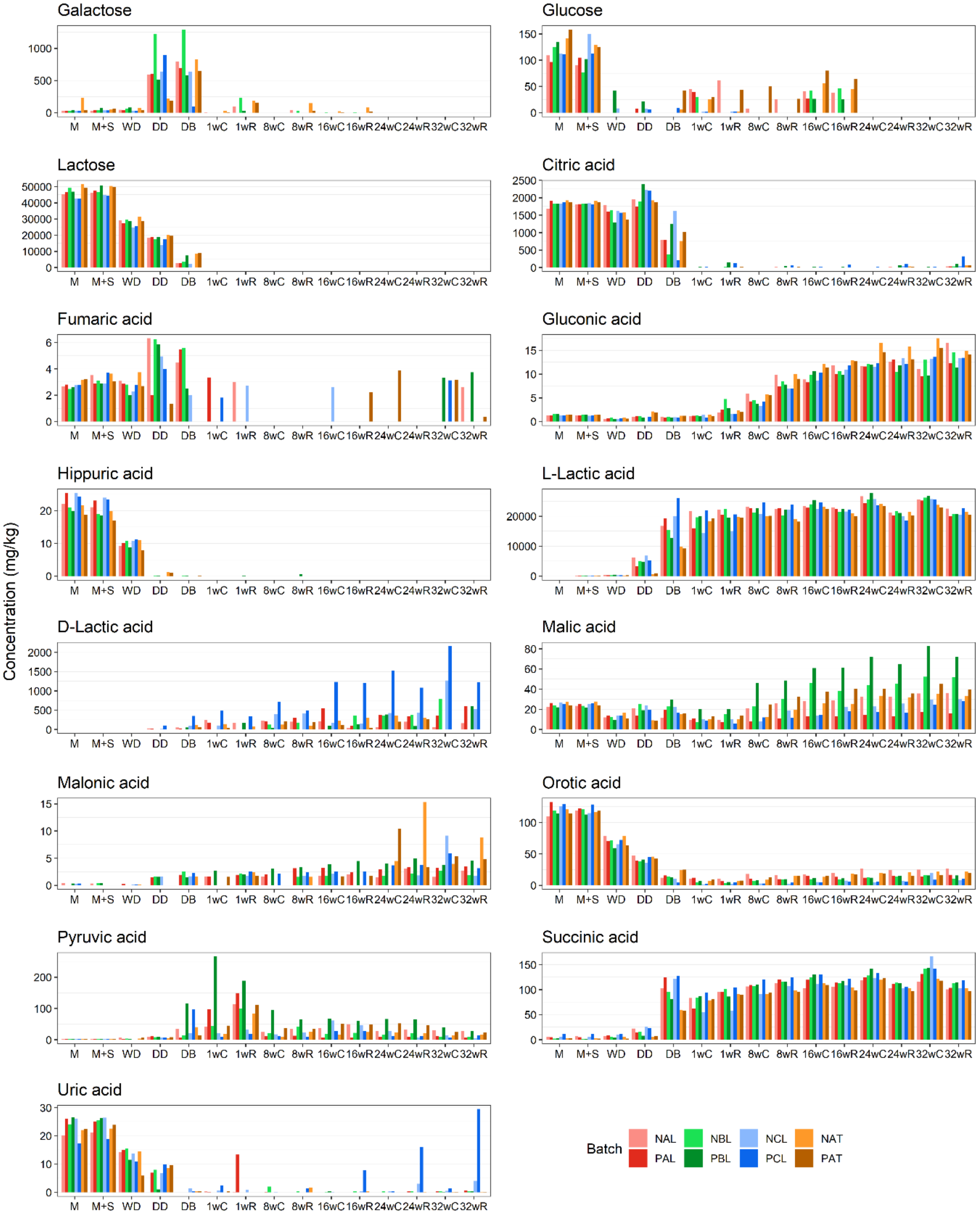

Figure S3. Dynamics of the concentrations of carbohydrates and organic acids during the six Gouda cheese production batches without and with the *Lacticaseibacillus paracasei* LP46 adjunct [NAL (light red), PAL (red), NBL (light green), PBL (green), NCL (light blue), and PCL (blue)], and during the two Gouda cheese production batches without and with the *Tetragenococcus halophilus* TH63 adjunct [NAT (orange) and PAT (brown)]. The codes of the production batches are as in the legend of Figure 7. The codes used for the time points are as explained in Table 1. C, core; R, rind.

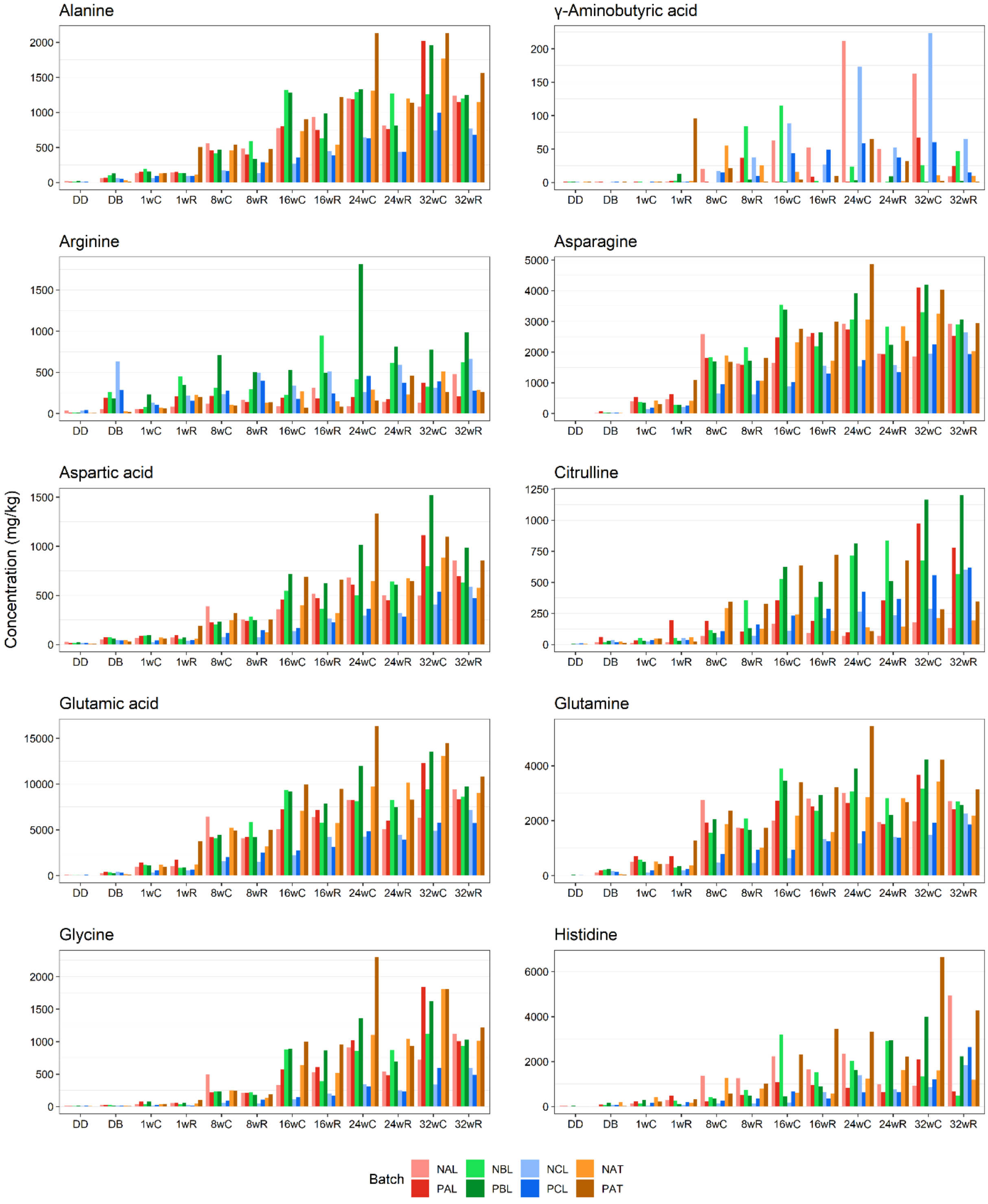

Figure S4. Dynamics of the concentrations of amino acids and biogenic amines during the six Gouda cheese production batches without and with the *Lacticaseibacillus paracasei* LP46 adjunct [NAL (light red), PAL (red), NBL (light green), PBL (green), NCL (light blue), and PCL (blue)], and during the two Gouda cheese production batches without and with the *Tetragenococcus halophilus* TH63 adjunct [NAT (orange) and PAT (brown)]. The codes of the production batches are as in the legend of Figure 7. The codes used for the time points are as explained in Table 1. C, core; R, rind.

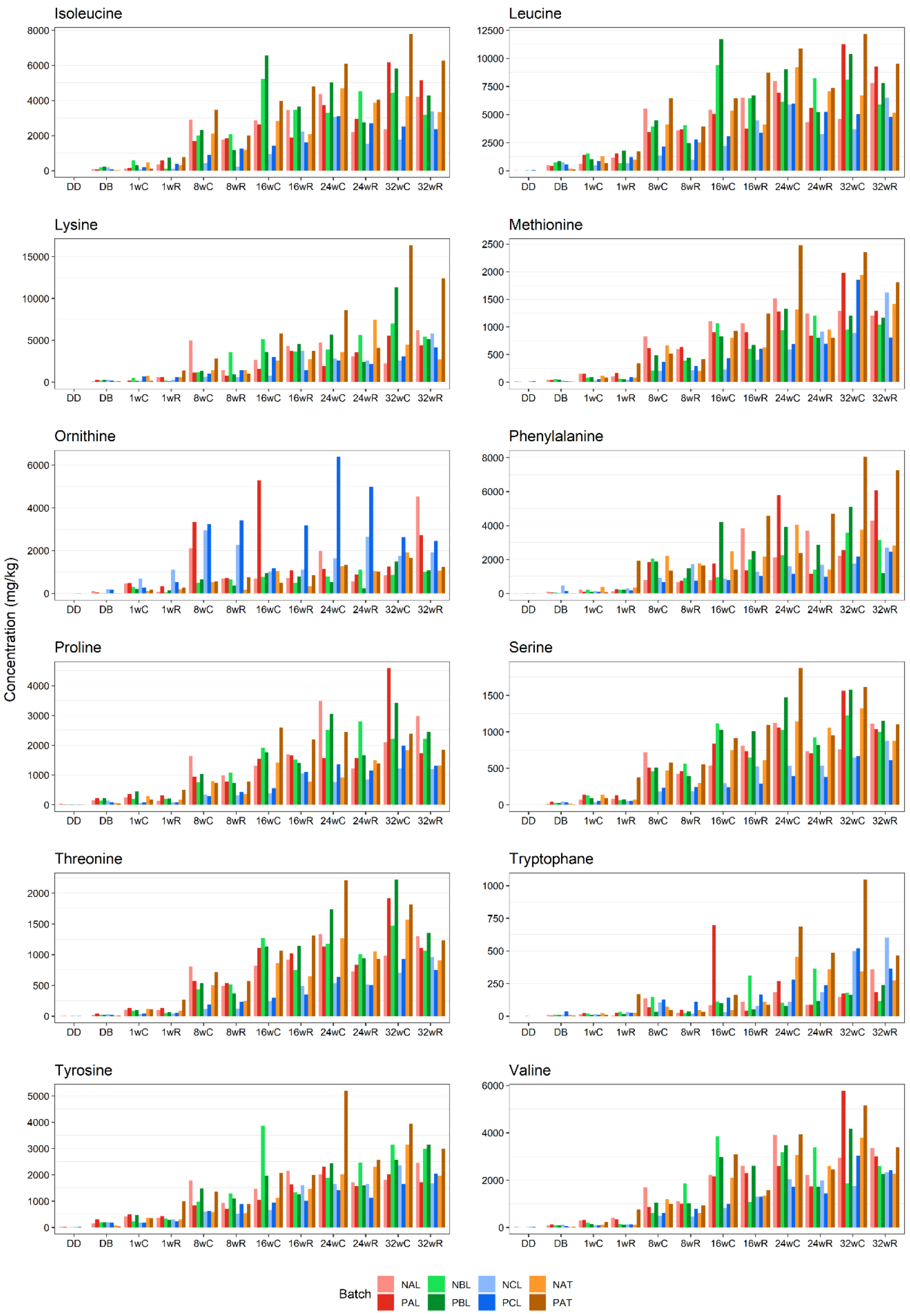

Figure S4. *Continued*.

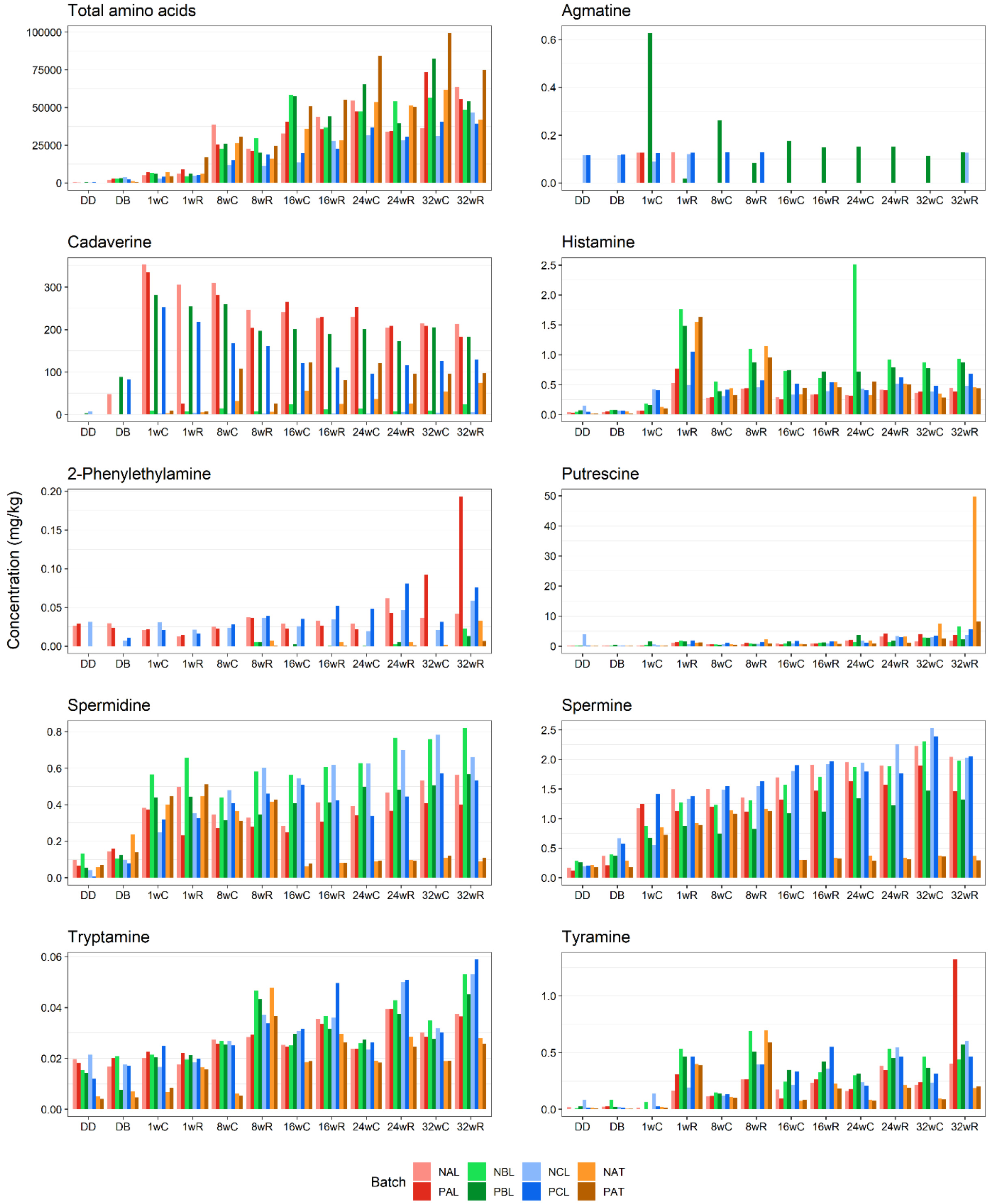

Figure S4. *Continued.*

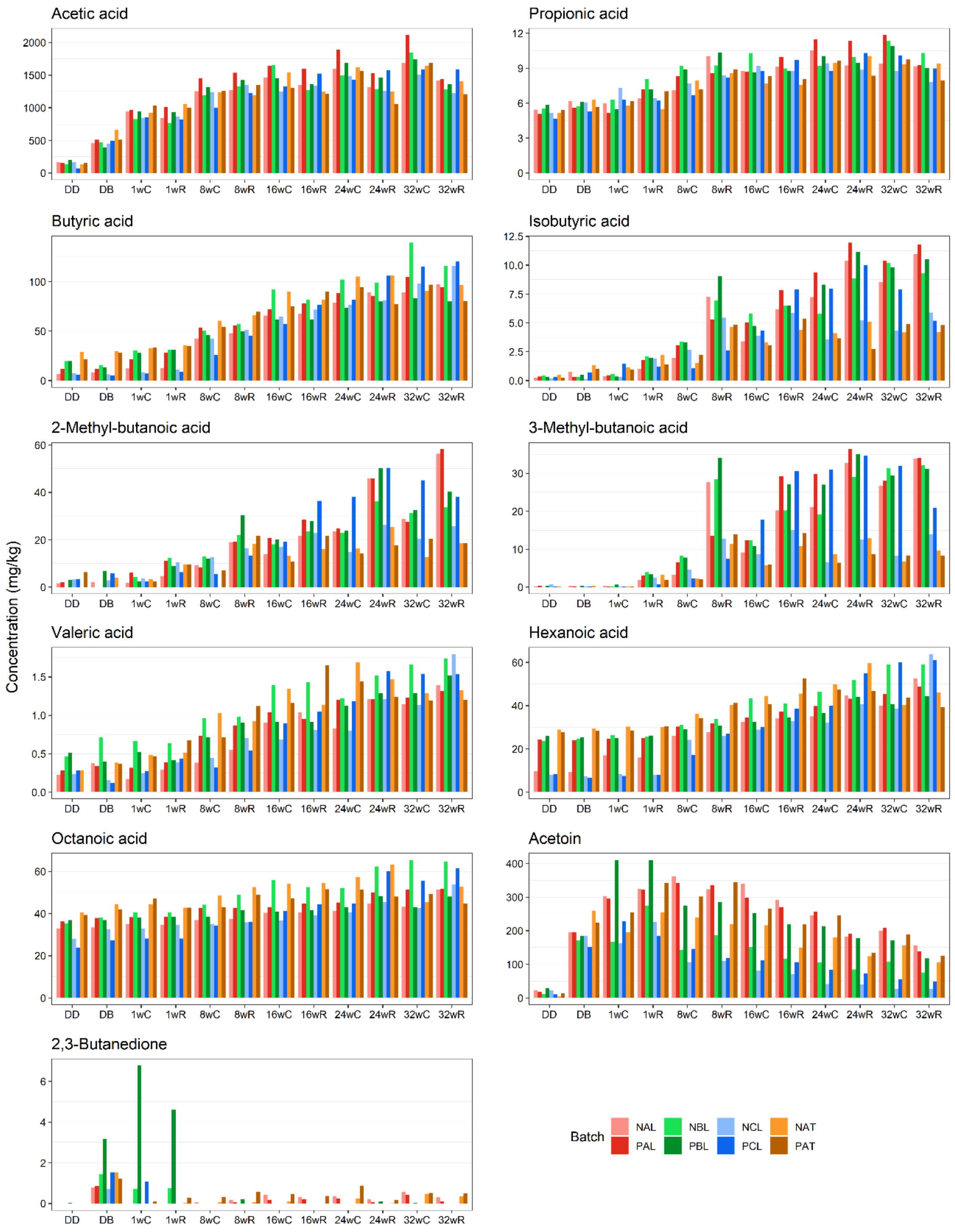

Figure S5. Dynamics of the concentrations of short-chain fatty acids and volatile organic compounds during six Gouda cheese production batches without and with the *Lacticaseibacillus paracasei* LP46 adjunct [NAL (light red), PAL (red), NBL (light green), PBL (green), NCL (light blue), and PCL (blue)], and during the two Gouda cheese production batches without and with the *Tetragenococcus halophilus* TH63 adjunct [NAT (orange) and PAT (brown)]. The codes of the production batches are as in the legend of Figure 7. The codes used for the time points are as explained in Table 1. C, core; R, rind.

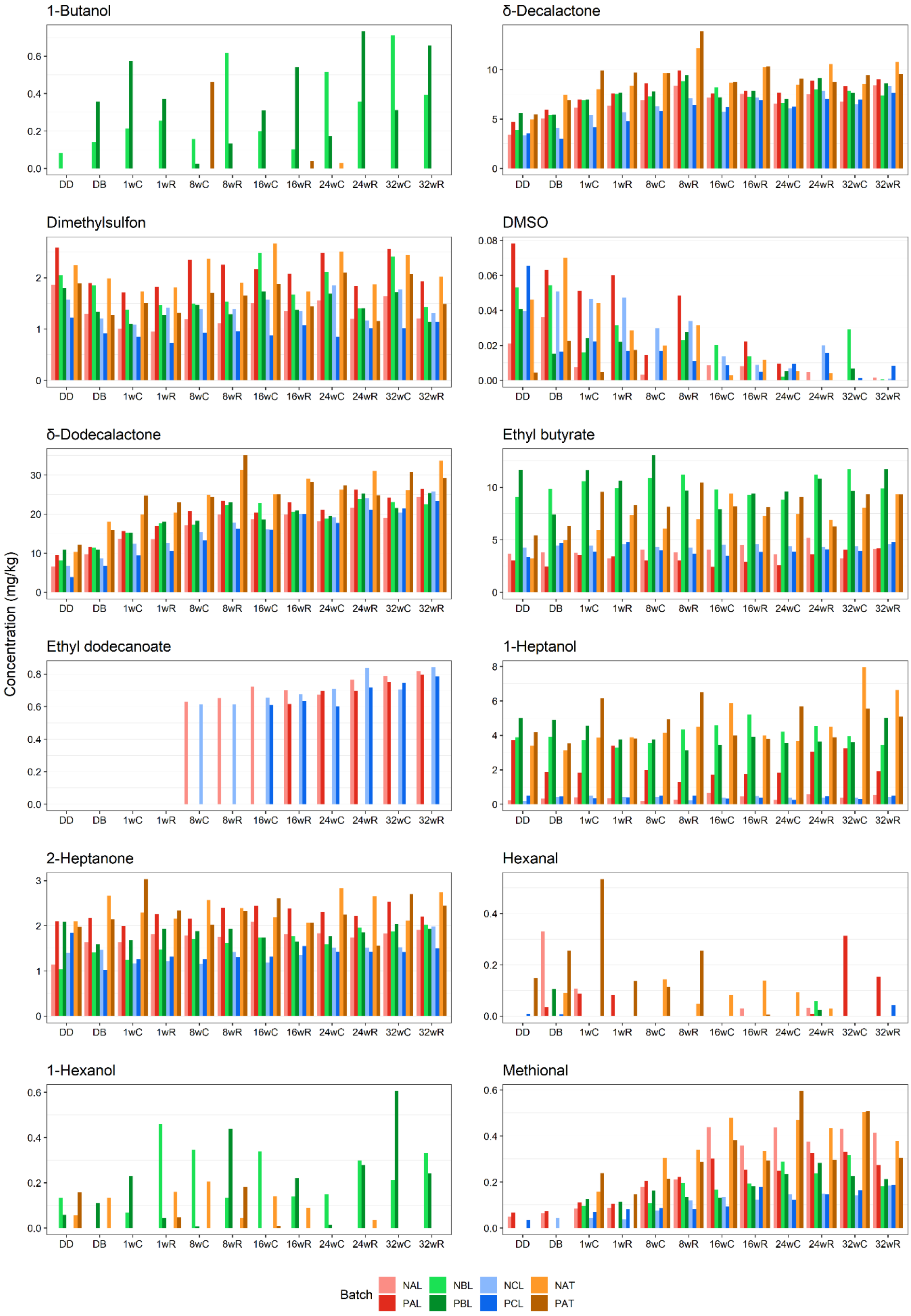

Figure S5. *Continued.*

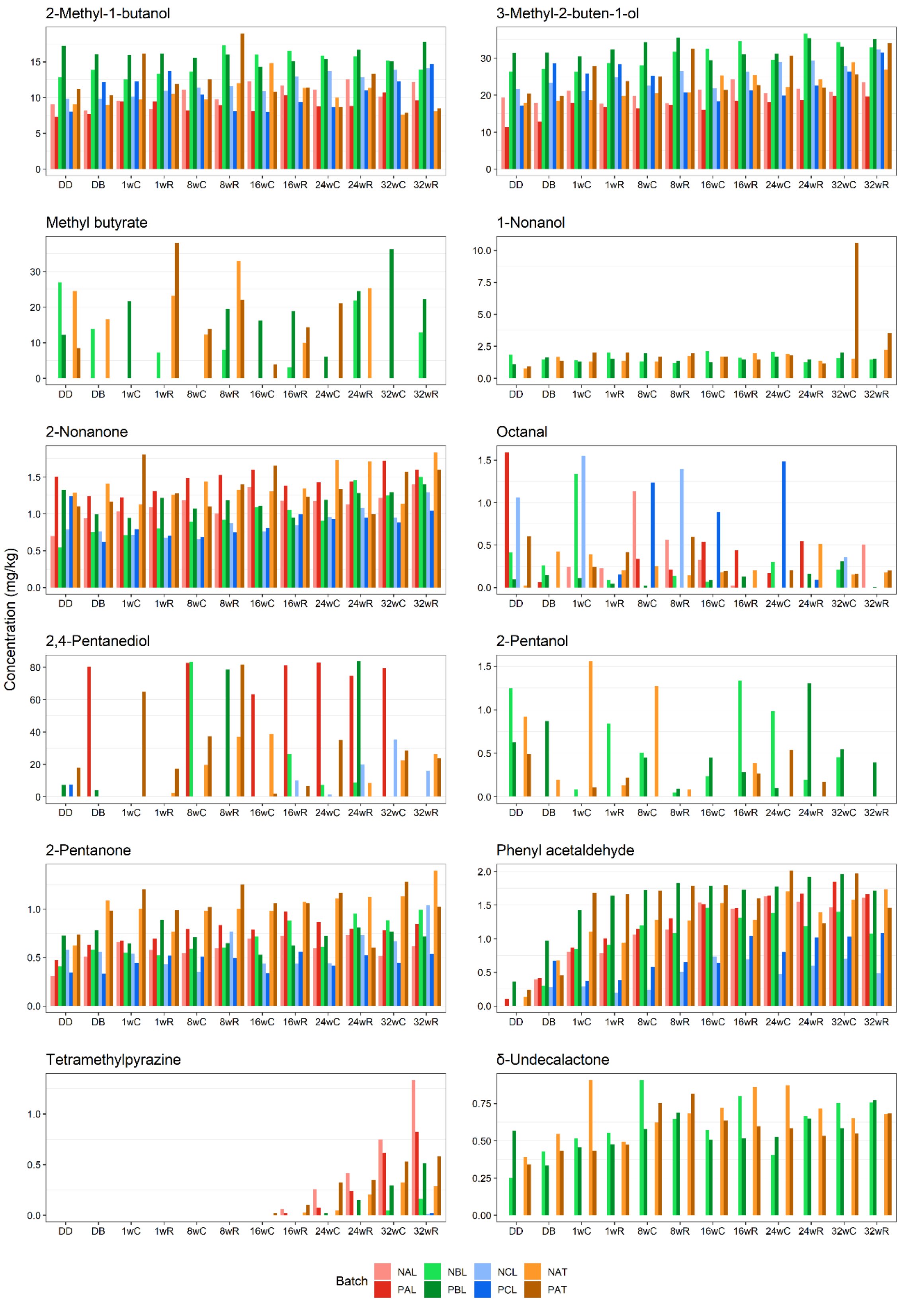

Figure S5. *Continued.*

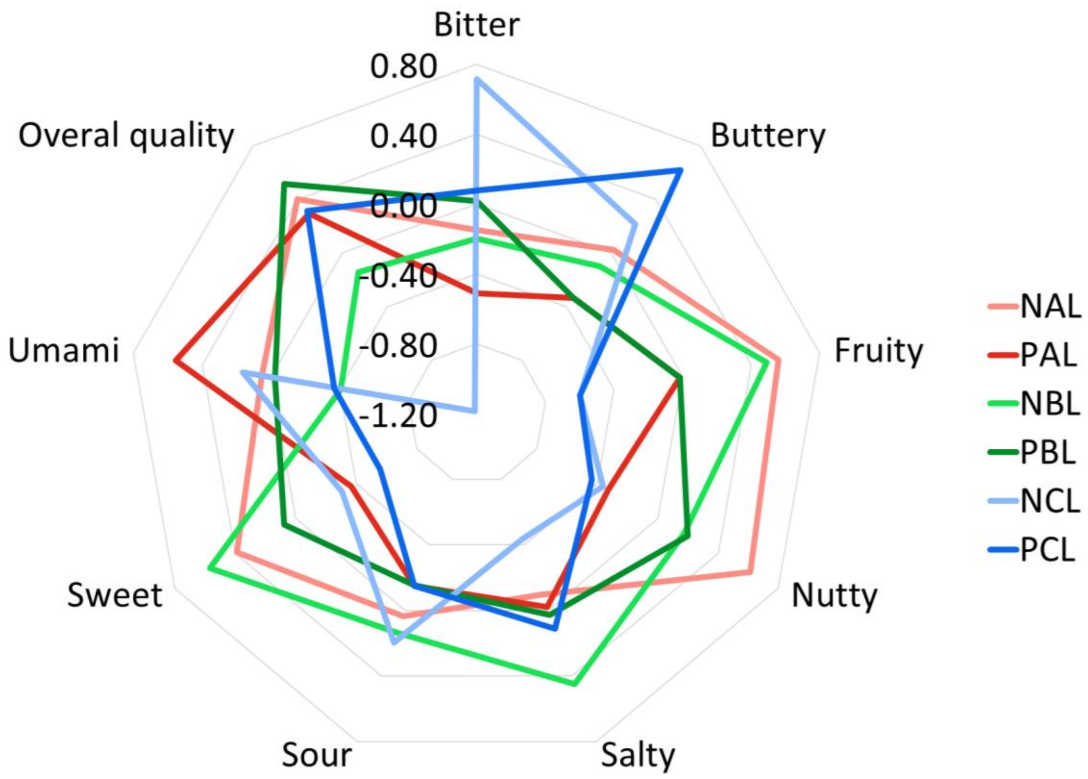

Figure S6. Z-scores of the organoleptic descriptors of the six Gouda cheese production batches without and with the *Lacticaseibacillus paracasei* LP46 adjunct [NAL (primary starter culture mixture A, without adjunct; light red), PAL (primary starter culture mixture A with adjunct, red), NBL (primary starter culture mixture B without adjunct, light green), PBL (primary starter culture mixture B with adjunct, green), NCL (primary starter culture mixture C without adjunct, light blue), and PCL (primary starter culture mixture C with adjunct, blue) after 32 weeks of ripening.
